# Characterization of the Edge States in Colloidal Bi_2_Se_3_ Platelets

**DOI:** 10.1021/acs.nanolett.3c04460

**Published:** 2024-04-16

**Authors:** Jesper R. Moes, Jara F. Vliem, Pedro M. M. C. de Melo, Thomas C. Wigmans, Andrés R. Botello-Méndez, Rafael G. Mendes, Ella F. van Brenk, Ingmar Swart, Lucas Maisel Licerán, Henk T. C. Stoof, Christophe Delerue, Zeila Zanolli, Daniel Vanmaekelbergh

**Affiliations:** 1 Debye Institute for Nanomaterials Science, Utrecht University, Princetonplein 1, 3584 CC Utrecht, The Netherlands; 2 Institute for Theoretical Physics and Center for Extreme Matter and Emergent Phenomena, Utrecht University, Princetonplein 5, 3584 CC, Utrecht, The Netherlands; 3 Université de Lille, CNRS, Université Polytechnique Hauts-de-France, Junia, UMR 8520-IEMN, F-59000 Lille, France

**Keywords:** Edge state, Bismuth selenide nanoplatelets, Scanning tunneling spectroscopy, Topological insulator, Density functional theory, Quantum spin Hall insulator

## Abstract

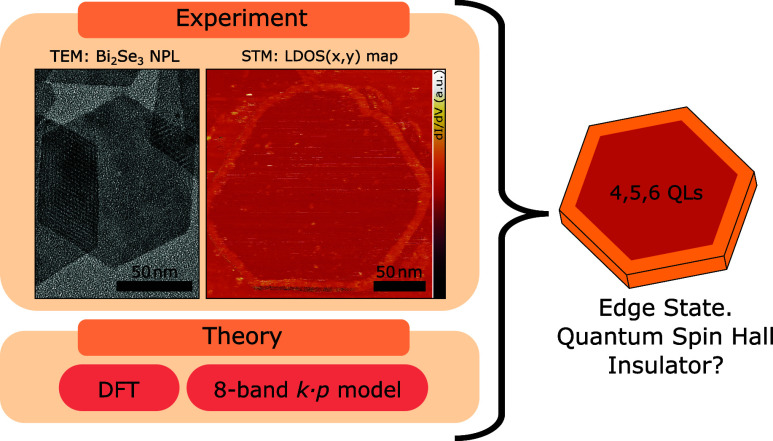

The remarkable development of colloidal nanocrystals with controlled dimensions and surface chemistry has resulted in vast optoelectronic applications. But can they also form a platform for quantum materials, in which electronic coherence is key? Here, we use colloidal, two-dimensional Bi_2_Se_3_ crystals, with precise and uniform thickness and finite lateral dimensions in the 100 nm range, to study the evolution of a topological insulator from three to two dimensions. For a thickness of 4–6 quintuple layers, scanning tunneling spectroscopy shows an 8 nm wide, nonscattering state encircling the platelet. We discuss the nature of this edge state with a low-energy continuum model and ab initio GW-Tight Binding theory. Our results also provide an indication of the maximum density of such states on a device.

In the last three decades, the field of colloidal nanocrystals has witnessed a remarkable development toward a versatile platform, offering nanocrystals of various chemical families with controlled shape, size, and surface chemistry.^1−3^ Today, brightly emitting semiconductor nanocrystals are successfully applied in the optoelectronic industry.^4−7^ Their success urges the question of whether colloidal nanocrystals could be of use for even more demanding purposes related to the emerging field of quantum materials, where the coherence of electronic states is the main focus.

In two-dimensional (2D) quantum spin Hall insulators (QSHIs), the interior of the material is insulating with a spin–orbit driven inversion of the valence and conduction bands. Due to the bulk-boundary correspondence, this results in a protected state at the edge of the 2D crystal with spin–momentum locking, crossing the inverted gap.^8,9^ The helicity impedes backscattering from lattice vibrations and nonmagnetic impurities. Therefore, QSHIs may form the basis for nondissipative information transfer devices with a considerable reduction of energy consumption. Moreover, a QSHI can be converted into a topological superconductor by proximity engineering.^10−13^ Such superconductors are of high scientific interest for advanced quantum computing.^10,14,15^

Bulk, three-dimensional Bi_2_Se_3_, is a well-known topological insulator^16−19^ with a large inverted gap of 200–300 meV and 2D Dirac-cone surface states that have been fully characterized with angle-resolved photoemission spectroscopy (ARPES).^16^ Bi_2_Se_3_ is a layered material with an integer number of quintuple layers (QLs, see Figure 1), stacked with weak van der Waals interactions. But what happens if the thickness of the layered Bi_2_Se_3_ crystal is reduced to a small number^1−6^ of QLs? Does the three-dimensional topological insulator transform into a two-dimensional quantum spin Hall insulator, with helical quantum channels at the border of the crystal? This question is relevant not only from a purely scientific perspective but also for applications, as electrical devices with a scalable density of quantum channels will require crystals of precise thickness and lateral dimensions. A first indication is given by the 4-band ***k·p*** model and ab initio simulations of ref (20), predicting that 2D Bi_2_Se_3_ crystals with a reduced thickness (≤7 QLs) are topologically nontrivial and should therefore have a one-dimensional, helical quantum state (with a predicted width of roughly 5 nm) at the crystal edge. Moreover, ARPES results on laterally extended crystals of 1*–*6 QLs in thickness show that the topological surface states of the top and bottom become gapped due to hybridization,^18,21,22^ indicating the transition from three to two dimensions. However, the presence and character of the quantum channels that may reside at the edges of finite-sized 2D crystals have not been addressed experimentally.

**Figure 1:**
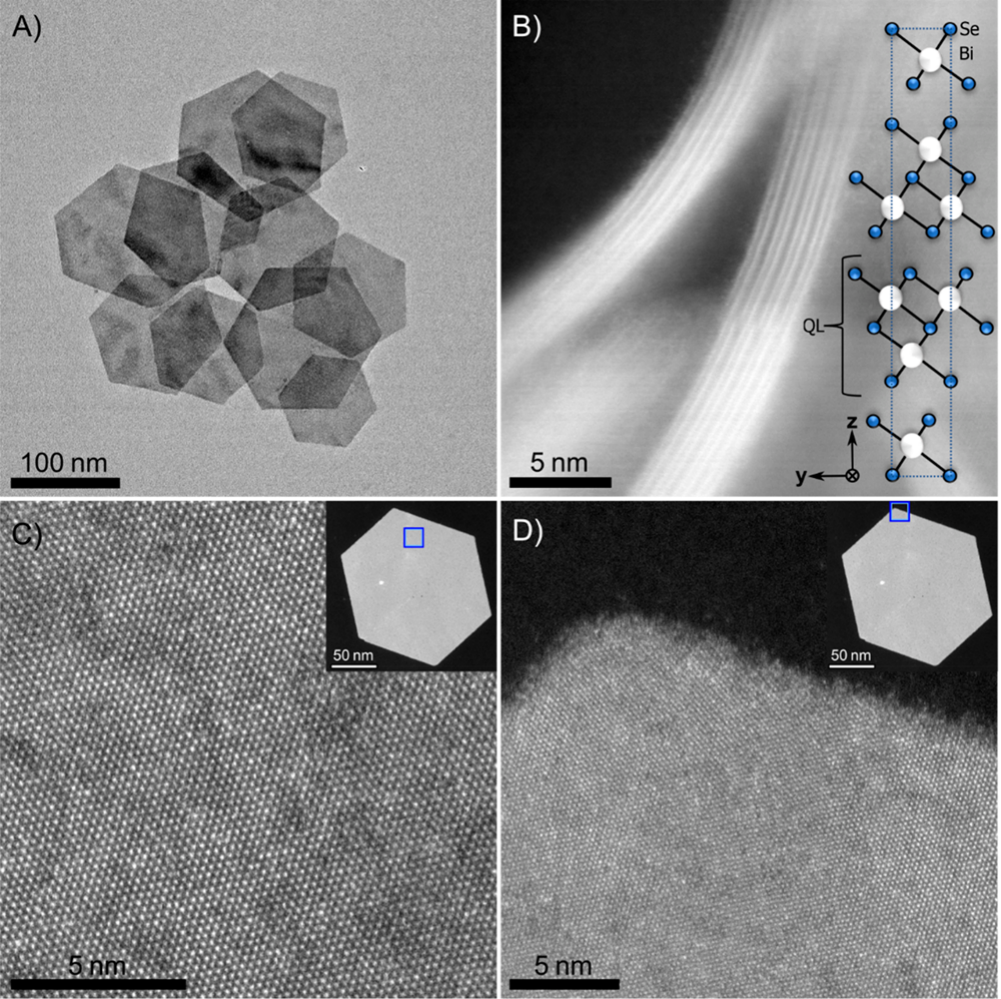
Structural characterization of colloidal Bi_2_Se_3_ NPLs. (A) TEM image of an ensemble of typical Bi_2_Se_3_ NPLs. (B) HAADF-STEM image with the viewing direction along the NPL, showing two NPLs consisting of 3 and 4 QLs, respectively. The two high intensity lines in each QL are due to the Bi columns (see inset). (C, D) High resolution HAADF-STEM images showing the high crystalline quality of the NPLs. The blue squares in the insets show the location at which the images were obtained.

Here, we use the virtues of colloidal chemistry to prepare 2D Bi_2_Se_3_ crystals with lateral dimensions in the 100 nm range and a uniform thickness of 1–6 QLs. These nanoplatelets (NPLs) can be expected to be strain-free, in contrast to larger crystals grown on a substrate.^23^ The limited dimensions of the platelets allow us to examine the crystal edges with scanning tunneling microscopy and spectroscopy (STM/STS). For each NPL, we measure the lateral dimensions, thickness, and density of states DOS (*E*, *x*, *y*) of the interior region and the edge. We find that edge states are absent for ultrathin (i.e., 1–3 QLs) platelets, while we observe an 8–10 nm wide channel at the perimeter of 4–6 QL crystals, with enhanced density-of-states over an energy region of several hundreds of meV, indicative of the quantum spin Hall effect. We have examined the nature of the edge states with a low-energy eight-band ***k·p*** model and first-principles calculations (GW-TB), from which we find evidence that the observed edge state may be a genuine helical quantum channel. Both models predict that the channels have a localization width in the 10 nm range, except very close to the Dirac point. These results indicate how close together one-dimensional quantum channels can be positioned in devices.

Bi_2_Se_3_ nanoplatelets were synthesized according to a procedure adapted from ref (24) that we have optimized to obtain highly crystalline platelets of relatively small lateral size (see Supporting Information, Materials and Methods). As shown in Figure 1A, the Bi_2_Se_3_ platelets have a hexagonal shape, uniform thickness, and a diameter of 166 ± 41 nm (Figure 1A,B and Figure S1A). Figure 1B shows a high-angle annular dark-field scanning transmission electron microscopy (HAADF-STEM) image of two NPLs of 3 and 4 QLs in the lateral viewing direction. The Bi layers (white atoms in the inset) can be observed as high-intensity lines. Figure 1C,D confirms the crystalline quality of the Bi_2_Se_3_ nanoplatelets, and energy dispersive X-ray spectroscopy ((S)TEM-EDX) measurements confirm the formation of Bi_2_Se_3_ (Figure S2). The thickness of the NPLs was determined using atomic force microscopy (AFM). As a single QL is 0.96 nm thick,^25^ the results indicate that most NPLs consist of 5–6 QLs (Figure S1B). We also prepared ultrathin (1–3 QLs) NPLs as shown in Figure S3.

Prior to STM/STS measurements, the NPL samples were treated to remove surfactants and organic contaminants as detailed in the Supporting Information. Subsequently, the platelets were cast on Au/Mica substrates and inserted in the scanning tunneling microscope in UHV. Before measuring, the samples were annealed at 393 K for 2 h to remove any residual organic contamination. Then, the sample was cooled to 4.5 K for UHV cryogenic scanning tunneling microscopy and spectroscopy. To investigate the effect of annealing on the nanocrystals, high resolution TEM was performed (see Supplementary Text and Figure S4). After annealing, we observed that Bi_2_Se_3_ material had been removed locally, which results in inclinations at the edges over a few nanometers. The results below show that the imperfections at the edge do not affect the interior band structure, nor the quality of the edge state.

We have investigated the electronic band structure of hexagonal Bi_2_Se_3_ platelets of 1–6 QLs in thickness by measuring the local DOS(E,x,y). For each Bi_2_Se_3_ platelet, the number of QLs can be retrieved by measuring the nominal height of the crystal at a constant tunneling current. The results are presented in the Supporting Information (Figures S5–S9). Here, we focus on a platelet with a thickness of 4 QLs; see Figure 2.

**Figure 2:**
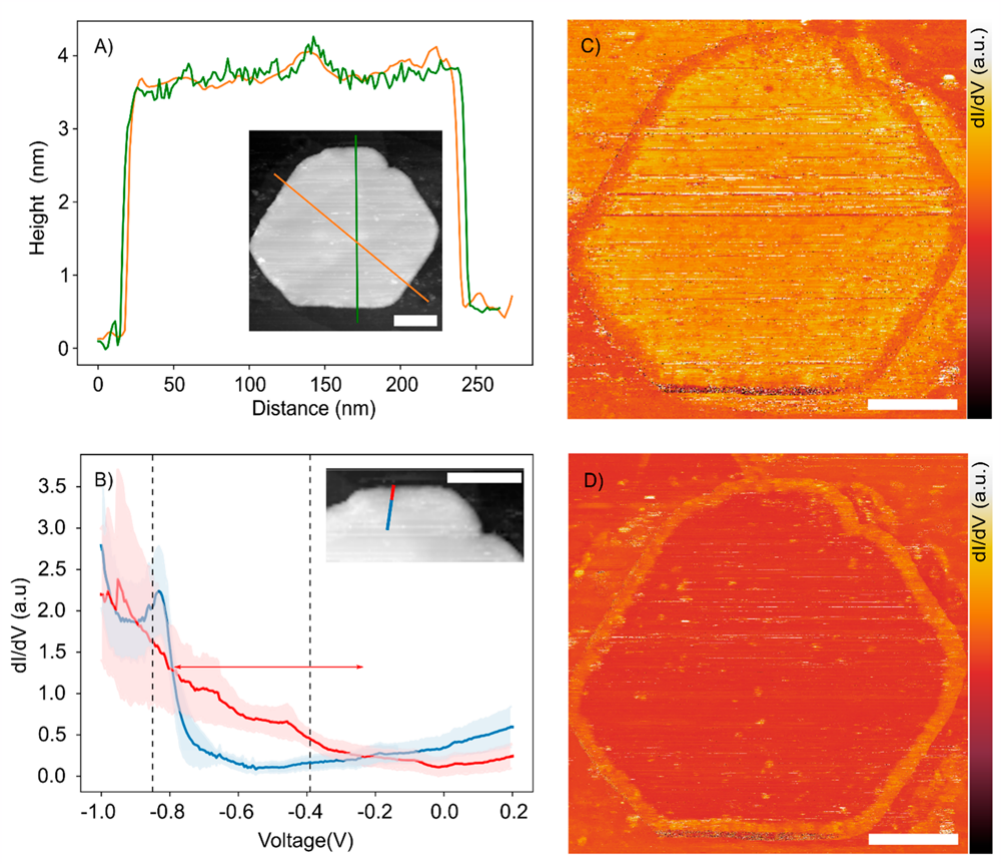
Characterization of the electronic states of a 4 QL thick Bi_2_Se_3_ platelet, in the interior and at the edge, with cryogenic scanning tunneling microscopy and spectroscopy. (A) Height profile of a single platelet on a flat Au substrate along the orange and green lines shown in the inset. The diameter of the 2D sheet is about 230 nm. The height profile shows this Bi_2_Se_3_ platelet consists of 4 QLs. (B) Scanning tunneling spectrum of the local DOS(*E*,*x*,*y*) in the interior and at the edge. The blue curve shows the spectrum averaged over 7 positions on the blue line of the inset,. The standard deviation is presented as a blue gloom. Similar spectra taken in the center of a platelet are presented in the Supporting Information, Figure S7. The red curve presents an average over 6 positions on the red line, in which the standard deviation is presented as a red gloom. This spectrum represents the edge state. The red arrow represents the energy region over which the density of states at the edge is larger than in the interior. The set point in the spectroscopy is 1 nA. (C) LDOS(x,y) map of the Bi_2_Se_3_ platelet acquired at a bias *V* of −0.85 V reflecting the top of the valence band. The edge region is uniformly dark, reflecting a lower DOS(*x*,*y*) at this potential. (D) LDOS(*x*,*y*) map of the Bi_2_Se_3_ platelet acquired at −0.39 V where the edge state is prominent. An 8–10 nm wide band of high density of states follows the edge of the crystal, including the edge imperfections. Scale bars are 50 nm. The set point in the maps is 0.5 nA.

Figure 2A presents the height profile of a platelet of 3.6 nm in height, i.e., a homogeneous thickness of 4 QLs and a diameter of roughly 220 nm. The microscopic STM image (inset) shows that the platelet has a hexagonal shape with some inclinations at the edge; see top left and right. We took the absence of irregular and spiky spectroscopic data as a measure of cleanliness of the surface of the Bi_2_Se_3_ crystal under investigation, as this led to reliable spectroscopic results in previous investigations of wet-chemically prepared nanocrystals, despite the lack of atomic resolved microscopy.^26^ We found that the spectra at different positions in the interior area of the platelet are all very similar, though with small quantitative differences. We therefore present position-averaged spectra for each platelet. Interestingly, the spectra taken in the interior area differ strongly from those taken in the last ∼10 nm from the edge. This is illustrated by the blue and red curves, respectively, in Figure 2B. The blue curve presents the d*I*/d*V* vs *V* spectra averaged over 7 positions on the blue line, typical for the DOS(*E*,*x*,*y*) in the interior area of the platelet. The standard deviation around the average is presented as a blue gloom. The strong rise in the DOS(*E*,*x*,*y*) negative of −0.8 V reflects the high density of states corresponding to the valence bands; vide infra. Positive of −0.8 V, above the valence bands, the DOS(*E*,*x*,*y*) is small but nonzero, and it slowly rises again at energies above −0.2 V. This low but nonzero density of states can be attributed to the hybridized surface states (see below). Generally, the spectra near (but not at) the edge are very similar to spectra taken in the very center of the platelet, as illustrated in Figure S10 for a 5QL platelet. Moreover, spectra measured on thin-film Bi_2_Se_3_ grown with precious gas phase methods^27−29^ are very similar to our interior area spectra. This shows that our Bi_2_Se_3_ platelets are large enough for them to be considered as genuinely 2D, and that the absence of atomic resolution in our measurements does not impede the spectroscopic investigation of the band structure.

When the STM tip approaches the crystal edge closer than 10 nm, shown as the red line in the inset, we observe a sudden change in the spectra. The averaged spectrum of 6 positions on this red line is shown as the red spectrum in Figure 2B, with the standard deviation as a red gloom. Typically, we observe a lower DOS(*E*,*x*,*y*) in the energy region of the valence band (*V* < −0.85 V), but a higher DOS in a broad region between −0.85 V and −0.2 V, indicated with the red arrow. This is also evident from the DOS(*x*,*y*) maps presented in Figure 2C,D. Panel C presents a map at −0.8 V over the entire crystal, illustrating an 8–10 nm wide band of lower density of states following the circumference of the crystal. Conversely, in the energy region above the valence band (Figure 2D) the situation is reversed with an 8–10 nm wide band highlighting a larger density of states. This band follows the perimeter of the entire crystal, including the two edge inclinations at the upper right and left. The DOS map at the crystal edge is very smooth, i.e., without density fluctuations. Below, we will argue that if backscattering occurs, it would lead to density fluctuations on a length scale around 10 nm, which would be clearly observable in the maps. The smooth appearance of the edge state in the DOS maps hence indicates that backscattering does not occur. A second example of a 4 QL crystal is presented in the Supporting Information, together with results on crystals of 1, 3, 5, and 6 QLs (Figures S5–S9). We find that Bi_2_Se_3_ platelets of 4–6 QLs show edge states very similar to those shown in Figure 2 (see Figures S7, S8, S9). The platelets of 1 and 3 QLs in thickness show a density of states uniform over the entire platelet from the interior to the edge, demonstrating the absence of an edge state (Figures S5 and S6).

Now, we argue that the edge states are not related to crystallographic disorder or a deviation in the chemical composition. Our TEM analysis (Figure 1 and Figures S1–S3) shows that the platelets are crystalline up to the last 1 nm region from the edge, where some disorder is visible. We also find that a combination of beam damage and annealing may cause removal of material at the edges in some NPLs (Figure S4). However, the edge states we observe are a factor of 10 wider than the 1 nm disordered region present in as-synthesized platelets, and they are uniform in width along the entire crystal. Furthermore, Figures 1 and S5–S9 show that the platelets on which we measured edge states have a uniform thickness. These are strong indications that crystal disorder cannot be related to the measured edge state. We also remark that all platelets are prepared with similar wet-chemical methods (see Materials and Methods) and have similar crystallographic quality. In contrast, edge states are observed only for platelets of 4–6 QLs, which again indicates that the edge states are not due to crystallographic or chemical disorder.

In summary, we observe a well-defined edge state of about 8 nm in width for Bi_2_Se_3_ platelets of 4–6 QLs in thickness, while this edge state is absent in platelets of 1–3 QLs in thickness.

We have examined the nature of the observed state using theoretical models on two levels of complexity. First, we have adapted the low-energy model presented by Zhang et al.^16^ to a more complete 8-band ***k·p*** model (Figure 3 and Figures S11 and S12), which allows us to calculate the Chern number per band and the  invariant; see Supplementary Text. It predicts that infinite 2D crystals of 3,4, and 5 QLs are topologically nontrivial  = 1) and hence quantum spin Hall insulators, while crystals of 1, 2, and 6 QLs are trivial. The 8-band model results in a Hamiltonian with two uncoupled and time-reversed 4-by-4 subspaces that we numerically solved. The solution for one subspace for a ribbon with long dimensions in the *x*-direction, width *L*_*y*_ of 100 nm, and thickness (*z*-direction) of 4 QLs is shown in Figure 3. The solution of the second subspace is simply the time-reversed version, presented in the Supporting Information, Figure S11. Figure 3A shows the low-energy bands related to the two inner (black) and the outer (surface, light blue) QLs. The Dirac line of the one-dimensional state at the edge is orange/red. Figure 3B presents the spin component of all bands averaged over the *z*-direction (solution of one subspace). For the edge state, we find that the z-averaged spin component in the (*x*, *y*) crystal plane is zero, but there is a clear *z*-averaged spin-polarization of 0.5 to 0.8 (in units of ℏ/2) in the *z*-direction, locked to the momentum. Importantly, the solution of the other subspace (Figure S11) shows the time-inversed spin polarization of −0.5 to −0.8 expected for the time reversed states. Both states together form the helical pair related to the predicted nontrivial topology for a Bi_2_Se_3_ crystal of 4 QLs. Figure 3A,B shows that the one-dimensional edge state connects to the valence and conduction bands of the inner QLs via the outer QL surface states. The calculated (L)DOS is presented in Figure 3C. The DOS(E) in the interior (blue) is comprised of the states of the inner and outer (surface) QLs, while the DOS(E) of the helical state at the edge is given in red. The DOS of the helical state is discernible over a relatively large energy region, although smaller than observed experimentally (see Figure 2). Figure 3D sketches the spatial characteristics of the helical edge state with momentum and z-averaged spin. The state extends in depth across 4 QLs (Figure S13), and has a small width of about 9 nm, which is very similar to the experimental result. We remark that the state becomes wider close to the Dirac point, and a slight hybridization of the left and right states can be observed if the ribbon width becomes too small (<100 nm). To make use of the nondissipative quantum channels in the most appropriate way in devices, the Fermi-level should therefore be distanced from the Dirac point.

**Figure 3:**
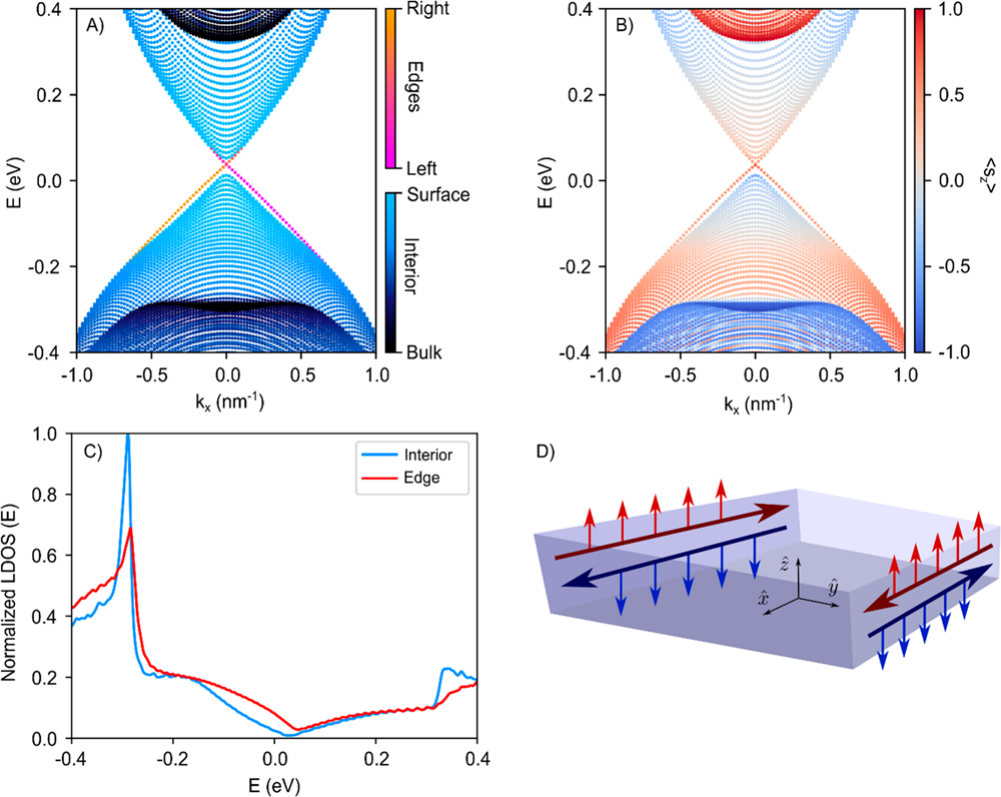
Theoretical analysis based on the low-energy eight-band ***k·p*** model for 2D Bi_2_Se_3_ ribbons, 4 QLs in thickness and 100 nm in width. (A) Solution of the upper 4 × 4 branch provides one of the two helical edge states (red/orange). The states related to the inner QLs are in black, and the (hybridized) top and bottom surface states are in light blue. (B) Same solution as in (A) but now presenting the spin-polarization ⟨*s*_*z*_⟩ of the states, averaged over the *z*-direction (hence over 4 QL thickness). The Dirac line represents one state of the helical pair at the edge with an average spin polarization between 0.5 and 0.8 (in units of ℏ/2). The time-reversed state is presented in the Supporting Information, Figure S12. (C) The resulting DOS for the ribbon of 4 QLs in thickness for the interior of the crystal (blue) and the edge (red). The edge state is discernible over a broad energy window, much broader than the inverted gap. (D) Scheme of the ribbon in real space with the calculated helical pair of edge states. The spin arrows reflect the projection on the *z*-axis (spin-polarization of 0.5–0.8 (in units of ℏ/2)) of the spin, averaged over the thickness of the platelet. Each state is spatially extended over about 8 nm inside the ribbon and is present across the 4 QLs (see Supporting Information, Figure S13). .

We present a similar characterization of the band structure for a ribbon of 2 QLs in thickness in Supporting Information, Figure S12. In this case, the bands are more strongly gapped and an edge state is absent (as  = 0). The same holds for a ribbon of a single QL. For 6 QLs, the ***k·p*** result also predicts  = 0, in disagreement with the observation of an edge state. We should remark here that the calculated trivial gap is tiny, and this disagreement should therefore be considered with care as the result is very sensitive to the bulk parameters used. Indeed, the first-principles theory (vide-infra) predicts  = 1, in agreement with our experiment result.

In addition to our 8-band ***k·p*** model, we used the GW approximation on top of first-principles density functional theory (DFT) to construct tight-binding (TB) models to calculate the band structure and DOS of infinite 2D Bi_2_Se_3_ crystals. The parameters were then transferred, without change, to TB calculations for ribbons of varying widths of up to 100 nm. For each thickness examined (1,2···6 QLs), the atomic structure of the Bi_2_Se_3_ crystal (atomic positions and cell) was thoroughly relaxed within the DFT framework, including spin–orbit coupling (see Table S1). For each of the relaxed structures, the Kohn–Sham wave functions were projected onto maximally localized Wannier functions from which an atomistic TB Hamiltonian capturing many-body exchange and correlation effects for the 2D crystal was constructed. The GW band structures for infinite crystals of 3–6 QLs are presented in Figure S14. Although calculation of the  invariant within the GW method is complicated due to the complex nature of the bands around Γ, we find that 2D Bi_2_Se_3_ with 1,2 and 3 QLs is trivial, while 4, 5, and 6 QLs are nontrivial; see Table S2. Figure 4A shows the GW band structure for an infinite 2D crystal with 4 QLs, which is in line with previous calculations^30^ and with reported ARPES results.^21^ The bandgap is inverted and has a value of 58 meV (see Table S3 for bandgaps of Bi_2_Se_3_ slabs of different thickness). We remark that the lowest doubly degenerate conduction and highest valence bands show spin–momentum locking (inset Figure 4A), reminiscent of their protected character in the 3D case. In Figure 4B,D, we present the band structures for Bi_2_Se_3_ ribbons of 4 QLs in thickness and 36 nm in width, with the infinite length in two orthogonal crystallographic directions resulting in a zigzag like edge (Figure 4C) or a straight edge (Figure 4E). In both cases there is an edge state connecting the valence- to the conduction bands, in agreement with both the nontrivial  invariant and the 8-band ***k·p*** model. Thus, our calculations show the quantum spin Hall effect for a fully relaxed atomic structure. We remark here that small residual strain can result in a small gap opening in the edge state dispersion, a situation very close to a trivial/topological phase transition (Figure S15). A similar situation has been previously reported, but remarkably, spin-momentum locking was still observed despite the gapping in the states.^31^ Lastly, Figure 4F shows the density of states in the interior (blue) and at the edges (solid and dashed red lines). Here too, we observe reasonable agreement with the experimental STS spectroscopy (Figure 2 and Supporting Information Figure S7).

**Figure 4:**
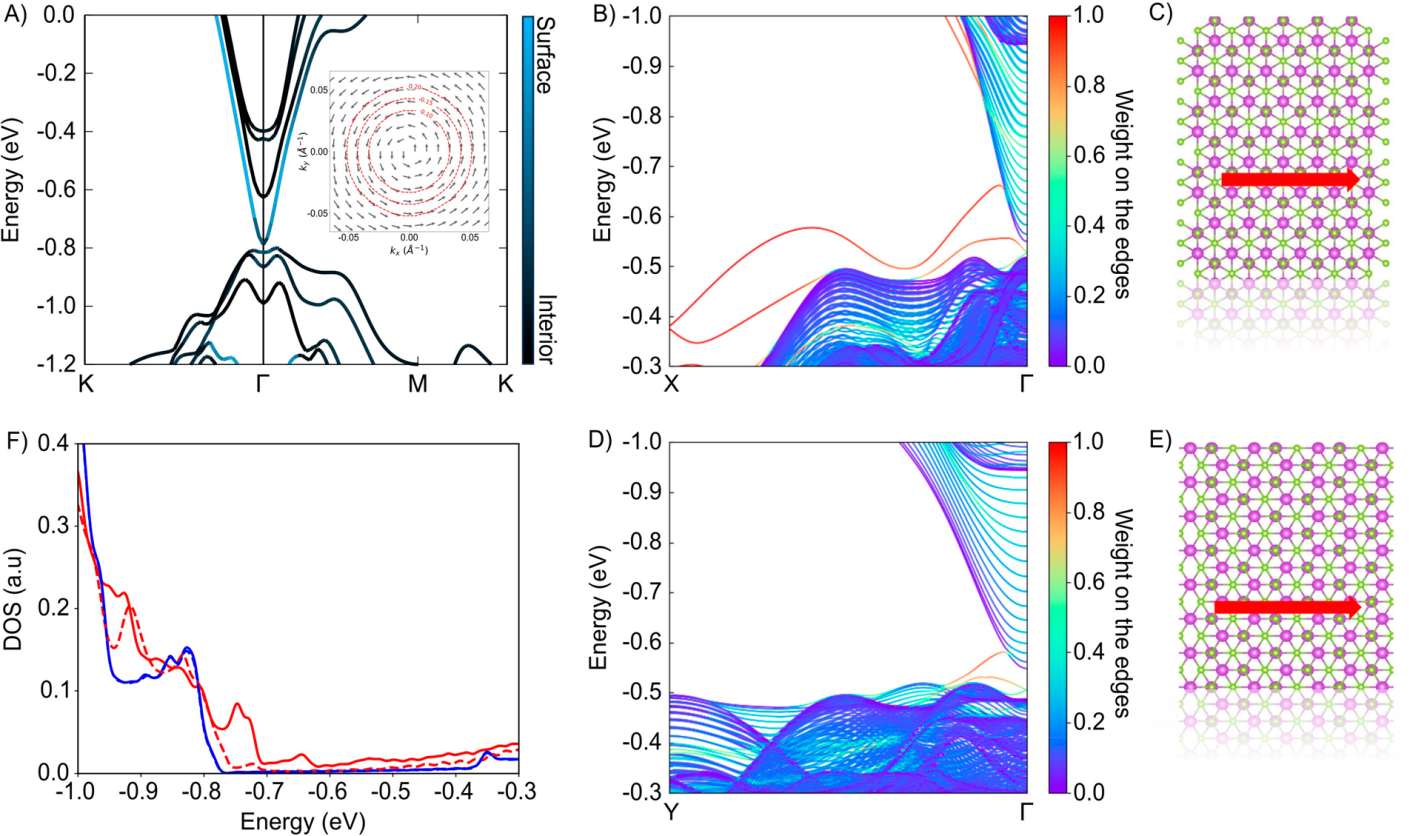
Theoretical analysis of the interior and surface states for a 2D infinite crystal, and edge states for 2D Bi_2_Se_3_ ribbons based on GW-TB calculations. (A) Electronic band structure for an infinite 2D Bi_2_Se_3_ crystal, 4QLs in thickness, computed within the GW approximation. The atomic structure is fully relaxed using first-principles DFT calculations including spin–orbit coupling. Interior states are in black, and surface states are in light blue. The inset shows the spin–momentum locking of one of the two lowest degenerate conduction bands (i.e., the blue surface state). The top of the valence band is set at −0.8 eV with respect to the Fermi level. (B, C) Band structure along the Γ–X line for a 4 QLs ribbon of 36 nm in width. The ball-and-stick model shows the termination (zigzag like) of the upper edge with the momentum for one of the helical states. Valence and conduction bands are connected by an edge state (red) in line with  = 1. (D, E) Similar, but now for a ribbon cut in the direction perpendicular, to (C). Valence and conduction bands are connected by an edge state (red) in line with  = 1. (F) The GW-TB calculated density of states in the interior of the ribbon (blue) with a peak corresponding to the top of the valence band (set at −0.8 eV with respect to the Fermi level) and showing the increasing density of states corresponding to the 4 lowest (doubly degenerate) conduction bands. Red-solid: density of states located at the edge with a zigzag like termination, corresponding to panels B and C. Red-dashed: density of states at the edge corresponding to the ribbon in panel E. Note that the experimental edge state width (10 nm) is broader than the ball-and-stick schemes shown in panels C and E.

Taking the results of the 8-band ***k·p*** model for k-values between 0.2–0.5 nm^–1^ (see Figure 3) we expect, in case of impurity scattering, oscillatory patterns on a length scale roughly between 10 and 30 nm, while the first-principles theory (Figure 4) predicts a length scale of 2.5–8.8 nm. Hence, in the case of backscattering, density-of-state oscillations on such length scales should be clearly observable in the platelets that we study. Indeed, such quasi-particle interference in thin crystals of Bi_2_Se_3_ has been observed, specifically for gapped surface states on step edges, see references (27) and (29). In our measurements, however, we did not observe such patterns. A systematic study of backscattering due to a magnetic impurity in a one-dimensional topological edge state has been presented in ref (32). It must be noted that the absence of quasi-particle interference is not an absolute proof for the nontrivial character of the edge state, as other factors, such as a short coherence length, could play a role. We additionally observe that the measured edge state in our NPLs remains unchanged under an external magnetic field up to 1 T (Figures S16 and S17 and Supplementary Text). Such resilience under an external field has been reported for other systems as well.^33−40^ Furthermore, the predictions of a helical edge state by the 8-band ***k·p*** model and the ab initio calculations provide a strong indication that the edge state that we observe in 2D Bi_2_Se_3_ platelets of 4,5 and 6 QLs is nontrivial.

The results presented here contribute to the understanding of the electronic topology in the evolution from a three-dimensional to a two-dimensional system. Using the virtues of colloidal chemistry, self-standing (thus strain-free) two-dimensional crystals of Bi_2_Se_3_ with atomically precise thickness and finite lateral dimensions were prepared and characterized with cryogenic scanning probe spectroscopy complemented with a theoretical analysis. For Bi_2_Se_3_ crystals of 4–6 QLs in thickness, we observe a one-dimensional 8–10 nm wide channel at the crystal edge that is absent in thinner crystals. We investigated the nature of this edge state with a continuum model and advanced ab initio calculations. The resemblance between the theoretical predictions for 2D Bi_2_Se_3_ of 1–6 QLs in thickness and the results of scanning tunneling microscopy and spectroscopy experiments form a strong indication that the edge state that we observe is a helical quantum spin Hall state. We emphasize that while our colloidal Bi_2_Se_3_ crystals provide an excellent model system for this fundamental study, growth from the gas phase combined with lithography will be required to create devices based on the quantum spin Hall effect.

## Supporting Information Available

Materials and Methods containing extended explanation on the synthesis procedure, characterization apparatus, and modeling of the band structure using DFT and G_0_W_0_; Supplementary Text containing information on the effect of annealing on the NPLs, explanation on the extension of the four-band ***k·p*** model into the eight-band ***k·p*** model, and motivation for measurements under external magnetic field (PDF)
Figures S1–S4 showing characterization of the Bi_2_Se_3_ nanoplatelets using AFM, EDX, and HAADF-STEM; Figures S5–S10 showing STM/STS data on Bi_2_Se_3_ nanoplatelets with thicknesses 1–6 QLs; Figures S11–S13 showing simulated data on 2QL and 4QL NPL using the 8-band ***k·p*** model; Figure S14 showing band structures computed with DFT and GW; Figure S15 showing GW-TB calculations for an infinite crystal and a 2D (100 nm) ribbon; Figure S16 showing STM/STS data on Bi_2_Se_3_ nanoplatelet (4QLs) subjected to a 1 T magnetic field; Figure S17 showing STM/STS data on Bi_2_Se_3_ nanoplatelet (4 and 6QLs) subjected to a 0.5 T magnetic field (PDF)
Table S1 listing simulated cell lattice parameters after geometric optimization using spin–orbit coupling; Table S2 listing calculated  invariants using DFT and G_0_W_0_ correction; Table S3 listing calculated bandgaps for Bi_2_Se_3_ slabs of different thicknesses (PDF)

## Abbreviations

NPL: nanoplatelet
STM/STS: scanning tunneling microscopy/scanning tunneling spectroscopy
2D: two-dimensional
QSHI: quantum spin Hall insulator
QL: quintuple layer
(L)DOS: (local) density of states
TB: tight-binding
DFT: density functional theory
HAADF-STEM: high angle annular dark field scanning transmission electron microscopy
ARPES: angle resolved photoemission spectroscopy
UHV: ultrahigh vacuum
AFM: Atomic force microscopy
EDX: energy dispersive X-ray spectroscopy
