# Supplementary material for: Characterization of the Edge States in Colloidal Bi2Se3 Platelets
Source: Nano Lett. 2024 Apr 16;24(17):5110–6. doi: 10.1021/acs.nanolett.3c04460 (PMC11066965; doi:10.1021/acs.nanolett.3c04460)
Supplement: Supplementary file 1 — nl3c04460_si_001.pdf [file nl3c04460_si_001.pdf]

Characterization of the edge states in colloidal Bi_2Se_3 platelets

*Jesper R. Moes[1]†, Jara F. Vliem[1]†, Pedro M. M. C. de Melo[1], Thomas C. Wigmans[1], Andrés R. Botello-Méndez[1], Rafael G. Mendes[1], Ella F. van Brenk[1], Ingmar Swart[1], Lucas Maisel Licerán[2], Henk T. C. Stoof[2], Christophe Delerue[3], Zeila Zanolli[1], *Daniel Vanmaekelbergh[1]*

ASSOCIATED CONTENT

Materials and Methods

Supplementary text

Materials and Methods

Chemicals

Na_2SeO_3 (99%, Aldrich), polyvinylpyrrolidone (Mw = 10,000, Aldrich), ethylene glycol (anhydrous, 99.8%, Aldrich), $Bi(NO_3)_3 \cdot 5H_2O$ (99,999%, Aldrich), Bi_2O_3 (>99%, J.T. Baker), ethanol (anhydrous 99.8%, VWR chemicals), acetone (anhydrous 99.8%, VWR chemicals), acetonitrile (anhydrous, 99.8%, Aldrich).

Synthesis procedure

Bi_2Se_3 nanoplatelets (NPLs) were synthesized according to a procedure adapted from Zhang *et al* (2011) (*1*). During a typical synthesis, 0.05 g of sodium selenite, 0.22 g polyvinylpyrrolidone (PVP) and 9.5 mL ethylene glycol are mixed in a 50 mL roundbottom flask. This mixture is degassed at the schlenkline for 15 minutes at room temperature. After degassing, the colorless solution is heated to 50 ^{o}C to dissolve all sodium selenite, followed by heating to 190 ^{o}C under N_2. This induces a gradual color change from yellow at 120 ^{o}C, to orange (130 ^{o}C), and finally dark red (180 ^{o}C). Once the mixture has reached 190 ^{o}C, a Bi precursor injection solution (0.2 g $Bi(NO_3)_3 \cdot 5H_2O$ in 1 mL ethylene glycol) is heated under N_2 on a 200 ^{o}C heating plate for 30 seconds after its color changed to turbid white, which typically occurs at 140 ^{o}C. 0.5 mL of this Bi precursor is then injected into the selenium mixture. After leaving the NPLs to grow for 10 minutes at 190 ^{o}C, the reaction mixture is cooled to room temperature using a water bath. The black product is transferred to a scintillation vial in a glovebox and washed by addition of acetonitrile and ethanol or acetone, followed by centrifugation. The supernatant is discarded and the black precipitate is redispersed in 10 mL ethanol. To prevent degradation, the samples were

stored in a glovebox under N_2 atmosphere. Ultra-thin (1-2 QL) platelets were prepared by using bismuth oxide instead of $Bi(NO_3)_3 \cdot 5H_2O$.

Prior to high resolution STEM measurements and STM/STS measurements, the NPL samples were treated to remove surfactants and organic contaminants. Depending on the sample, either hydrazine hydrate ($N_2H_4 \cdot xH_2O$ 50-60%, Aldrich) or oleylamine (70%, Aldrich) was added to strip off PVP ligands or facilitate additional washing of the NPLs. During a typical treatment, 1 mL of hydrazine hydrate or oleylamine was added to 1 mL NPL dispersion and the resulting mixture was shaken vigorously. The NPLs were subsequently isolated by centrifugation and redispersed in 1 mL ethanol. The resulting dispersion was washed a minimum of two times by addition of (a mixture of) ethanol and acetonitrile, followed by centrifugation and redispersion of the precipitate in 1 mL of ethanol. Samples with thin (<3 QL) platelets were used without additional treatment to prevent further clustering.

Characterization

A Talos F200X (S)TEM operating at 200 keV was used for imaging and for STEM-EDX analysis. An aberration-corrected Thermo Scientific Spectra 300 (S)TEM operating at 300 keV was used for high resolution imaging. TEM samples were prepared by drop-casting a diluted dispersion of NPLs on 200 mesh Formvar/carbon-coated Cu TEM grids (for low resolution measurements), 400 mesh ultra-thin (3 nm) carbon coated Cu TEM grids (high resolution measurements, or 200 mesh Quantifoil® holey carbon Cu TEM grids (side view of NPLs). Prior to high resolution measurements, the TEM samples were treated with ethanol and activated carbon according to a procedure reported by Li *et al.* (*2*). Thereafter, the TEM samples were

subjected to a final cleaning step by holding the grids in fumes of boiling acetone for 1 minute while drops of acetone condensed on the grid.

AFM measurements were carried out using a JPK Nanowizard II operating in intermittent-contact mode in ambient atmosphere. Bruker OTESPA-R3 (26 N/m) or MikroMasch ultrasharp SPM tips (CSC37/No Al, 0.3 N/m) were used to acquire images with typical set-points of 0.8 V. Samples were prepared by drop-casting or spin-coating 20 μL of a diluted NPL dispersion onto a freshly cleaved mica surface.

STM/STS measurements were obtained in a Scienta Omicron POLAR SPM Lab, operated at a base temperature of 4.5 K and with pressure in the 10^{-10} mbar range. The bias V was applied between the gold layer on the mica and the tip, with a negative bias meaning that the Fermi level of the gold substrate is higher than that of the tip. STM samples were prepared by diluting and sonicating the treated NPL dispersion, after which 10 μL was drop-cast or spin-coated on a Au(111)/MICA substrate purchased from Phasis Sàrl. Before measuring, the sample was annealed in the STM at 393 K for 1-2 hours.

STM topography images were acquired at a constant current of 0.2 nA and -1 V. Wave function mapping and differential conductance spectroscopy were performed using constant-height mode with a lock-in amplifier providing a 973 Hz bias modulation with an amplitude between 5 and 20 mV rms. Experimental data were analyzed with the SPM analysis software Gwyddion 2.54 and Python 3.7.

Ground state DFT simulations

Density Functional Theory (DFT) calculations were performed on several slabs of Bi_2Se_3, from one to six quintuple layers using the QuantumESPRESSO software package (*3*). Simulations were performed using norm-conserving fully relativistic pseudo-potentials from PseudoDOJO (*4*) with spin-orbit coupling, and GGA-PBE exchange-correlation functional (*5*). The in-plane lattice parameters were optimized for each slab until the maximum force per atom was less than 10^{-6} Ry/Bohr and the components of the stress tensor were below 10^{-4} kBar, within collinear-spin simulations. The separation distance between periodic replicas was 20 Å, and simulations were performed on a 12x12x1 k-point grid with a wave-function kinetic energy cutoff of 140 Ry. Long range van der Waals interactions were included via a Grimme D3 parametrization, but with no three-body interactions.

We investigated the nature of the electronic band structure by projecting each state on each individual atom and then adding the contributions of the atoms belonging to the same quintuple layer. This procedure reveals which electronic states are localized on the surface (*surface states*) or in the interior of the slab (*inner states*) as highlighted, for instance, in the GW band structure of figure 4A.

G_0W_0 simulations

The DFT energies obtained with QuantumESPRESSO where corrected using the quasi-particle full frequency G_0W_0 approximation with the Yambo package (*6*). The kinetic energy cutoffs were 45 Ha for the G-vectors in the Fast Fourier Transform and 4.250 Ha for the sum in G-vectors in the exchange and correlation self-energy. A total of 180 bands per layer were used in

the polarizability while 300 bands per layer were used in the correlation self-energy. The convergence of the sum over empty bands was accelerated by employing a terminator based on the Bruneval-Gonze technique. Finally, a truncation technique was used for the Coulomb potential to minimize the long-range interaction between replicas of the slabs, with the cutoff distance equal to $c - 2$ Bohr, where c is the out-of-plane lattice parameter for each slab. The computed DFT and GW band structures are shown in fig. S14 for 3-6QLs.

Extraction of Tight-Binding parameters from G_0W_0 and computation of the $\mathbb{Z}_2$ topological invariant from DFT and G_0W_0

The DFT ground state calculations were post-processed with the Wannier90 (*7*) package to obtain the Hamiltonian and wave-functions in the maximally localized Wannier function (MLWF) representation. The Wannier wave-functions and Hamiltonian were used to generate the Tight-Binding model Hamiltonian and Wannier charge centers which were then processed with WannierTools (*8*) to compute the topological invariant $\mathbb{Z}_2$ on DFT bands. The electronic bands and energies were parametrized up to a 36x36x1 k-point grid to ensure that the bands obtained from the Wannier model match those obtained directly from DFT. Only 30 bands per quintuple layer were parametrized and the window of frozen energies was kept at 1 eV around the Fermi level. The calculation of the $\mathbb{Z}_2$ topological invariant required a very dense k-sampling, depending on the number of QLs. For Bi_2Se_3 of 1QL and 2QL, a 15x15x1 k-sampling was sufficient to converge $\mathbb{Z}_2$. For 3 to 6QL, however, several hundreds to thousands of k points were needed to achieve convergence of $\mathbb{Z}_2$.

To confirm that the topological properties are preserved after the G_0W_0 corrections of the energy levels, we computed the $\mathbb{Z}_2$ topological invariant for the G_0W_0 energies. We followed the same procedure as in the DFT case, with the same convergence parameter, and processed the G_0W_0 energies with Wannier90 and WannierTools. In both DFT and G_0W_0 cases, the onset of the topological behavior is likely at 4 QL.

Supplementary Text

In-situ annealing of NPLs

Annealing of the NPLs in the STM is required to remove any remaining contamination and facilitate STM measurements. To investigate the influence of annealing on the NPLs, in-situ TEM experiments were performed using an aberration-corrected Thermo Scientific Spectra 300 (S)TEM operating at 300 keV. Parameters were chosen that resemble the conditions in the STM closely: 2 μL of a diluted NPL dispersion was drop-cast on a MEMS heating chip, after which the chip was heated to 393 K at a rate of 10 K/min. The electron beam was blanked during annealing to prevent beam damage. After 2 hours of annealing at 393K, high resolution HAADF-STEM images were collected (fig. S4).

While the NPLs retain their hexagonal shape, thinning of the crystals at predominantly the corners and edges is observed in many NPLs as shown in fig. S4a. Fig. S4B-D show a selection of NPL corners and edges at higher magnification. In some NPLs (fig. S4B) the crystal structure remains intact, but in most images a lower contrast can be observed at the corners of the NPLs compared to the center (fig. S4C,D). This suggests that the NPLs have lost material starting from

the edges and corners during annealing. We do note that the observed change in the NPL crystal structure appears to be escalated by the electron beam.

Modeling of the band structure

We use the four-band $\boldsymbol{k}\cdot\boldsymbol{p}$ Hamiltonian derived by Zhang *et al.* (*9*) for describing 3D Bi_2Se_3 around the 3D Γ point. In the basis $(|\mathrm{Bi}^+,\uparrow\rangle, |\mathrm{Se}^-,\uparrow\rangle, |\mathrm{Bi}^+,\downarrow\rangle, |\mathrm{Se}^-,\downarrow\rangle)$ it has the form

$$H(\boldsymbol{k},k_z) = \epsilon_0(\boldsymbol{k},k_z)\mathbb{I}_{4\times4} + \begin{bmatrix} \mathcal{M}(\boldsymbol{k},k_z) & A_1k_z & 0 & A_2k_- \\ A_1k_z & -\mathcal{M}(\boldsymbol{k},k_z) & A_2k_- & 0 \\ 0 & A_2k_+ & \mathcal{M}(\boldsymbol{k},k_z) & -A_1k_z \\ A_2k_+ & 0 & -A_1k_z & -\mathcal{M}(\boldsymbol{k},k_z) \end{bmatrix},$$

where $\boldsymbol{k} = (k_x, k_y)$ is the in-plane momentum, $\epsilon_0(\boldsymbol{k},k_z) = C + D_1k_z^2 + D_2\boldsymbol{k}^2$, and $\mathcal{M}(\boldsymbol{k},k_z) = M - B_1k_z^2 - B_2\boldsymbol{k}^2$. All parameters are fitted from the *ab initio* calculation of ref. *9* and their values are: $C = -0.0068$ eV, $M = 0.28$ eV, $A_1 = 0.22$ eV nm, $A_2 = 0.41$ eV nm, $B_1 = 0.10$ eV nm^2, $B_2 = 0.566$ eV nm^2, $D_1 = 0.013$ eV nm^2, and $D_2 = 0.196$ eV nm^2. It is important to note that our model does not take any further inputs, and the models for thin nanosheets will be derived directly from the above 3D bulk model.

The model is solved at the 2D Γ point ($k_x = k_y = 0$) in a slab geometry via the substitution $k_z \to -\mathrm{i}\partial_z$ and hard-wall boundary conditions $\Psi(z = \pm L_z/2) = 0$, with L_z the nanosheet thickness. As a result of the confinement in the z-direction we obtain an infinite set of transverse states, which at the 2D Γ point separately describe spin-up and spin-down electrons. Four of these states are surface states whose dispersions at large L_z come in the form of a gapless Dirac cone, but the cone becomes gapped at the small thicknesses of interest as a result of the

hybridization between states on the top and bottom surfaces. To obtain an accurate effective Hamiltonian, we project the Hamiltonian at finite $\boldsymbol{k}$ on a subset of these states. We take the four surface bands corresponding to the gapped Dirac cone and also the first set of four bulk bands closest to the Fermi level. The basis is ordered as follows:

$$[\varphi_\uparrow^S \quad \chi_\downarrow^S \quad \varphi_\uparrow^B \quad \chi_\downarrow^B \quad \varphi_\downarrow^S \quad \chi_\uparrow^S \quad \varphi_\downarrow^B \quad \chi_\uparrow^B],$$

where the z-dependent states φ and χ are defined in ref. *10* and the superscripts S and B refer to the surface and bulk bands, respectively. The resulting Hamiltonian is an 8×8 Hermitian matrix that decouples into two 4×4 subblocks as a combined effect of the time-reversal symmetry (TRS) and the mirror symmetry with respect to the xy-plane. The two subspaces are related by TRS, so here we present the Hamiltonian of only one of them, which reads

$$\begin{bmatrix} H_{SS} & H_{SB} \\ H_{SB}^\dagger & H_{BB} \end{bmatrix}.$$

Here,

$$H_{II} = \begin{bmatrix} E_0^I - D^I\boldsymbol{k}^2 + (M^I - B^I\boldsymbol{k}^2) & A^I k_- \\ A^I k_+ & E_0^I - D^I\boldsymbol{k}^2 - (M^I - B^I\boldsymbol{k}^2) \end{bmatrix}, \quad H_{SB} = \begin{bmatrix} ak^2 & bk_- \\ ck_+ & dk^2 \end{bmatrix},$$

where $I = S, B$, $k_\pm = k_x \pm \mathrm{i}k_y$, and all parameters $(E_0^I, D^I, M^I, B^I, A^I, a, b, c, d)$ depend on the thickness L_z and together encode the topology of the material. As mentioned above, these parameters are derived unambiguously from the initial set of bulk parameters. In previous works (*10,11*), only H_{SS} was considered as an effective Hamiltonian for thin films, but we find that this

approximation can only describe thicknesses of up to 3 QLs. We observe that for thicker nanosheets of 4, 5, and 6 QLs the bulk bands are required as well to give accurate results.

For the effective 8-band model we calculate the $\mathbb{Z}_2$ invariant ν by counting the number of pairs of zeros of the Pfaffian of the occupied subspace of the time-reversal matrix on a contour that encloses exactly half of our (infinite) Brillouin zone (*12*). Since the system has also inversion symmetry, this is the same as computing the product of parities of the Kramers pairs at the time-reversal invariant momenta (*13*), which in our continuum model are $|\boldsymbol{k}| = 0$ and $|\boldsymbol{k}| = \infty$.

We have also computed the energy spectrum of the 8-band model on a ribbon of 100 nm width. When the $\mathbb{Z}_2$ invariant is nonzero we find states localized at the edges whose dispersion lives in the energy gap and forms a Dirac point with linear dispersion. Due to finite-size effects the states hybridize slightly at the Dirac point and a small gap opens. When the $\mathbb{Z}_2$ invariant is trivial, no edge states are found. In figs. S11 and S12 we show the spectrum for 4 QLs (topological) and 2 QLs (trivial) respectively to illustrate this point.

In fig. S13 we also show the local density of states (LDOS) of the edge modes in the case of 4 QLs. As can already be seen in the fig. S11, they span an energy range of about 200 meV. Furthermore, their penetration into the sample for a width of 100 nm is about 9 nm, in very good agreement with the experimental findings.

Measurements under external magnetic field

Because time-reversal symmetry is a requirement for the QSHI phase (*14*), a magnetic field perpendicular to the 2D crystal breaks TRS and should thus be incompatible with helical quantum channels at the edge of the crystal. However, recent theory and experimental results

have shown that edge states remain, even under a considerable perpendicular magnetic field (*15-18*). For the case of the InAs/GaSb system, a 2D QSHI (*19-22*), it has been shown theoretically (*21*) and experimentally (*22*) that the edge states are resilient and show (nearly) quantized conductance up to high magnetic fields of 10 T. We have therefore studied the interior- and edge states of 2D Bi_2Se_3 crystals under perpendicular fields of 0.5 and 1 T. The results for a platelet with 4 QLs (the same as presented in Fig. 2) are shown in fig. S16, and more results for plates with 4 and 6 QLs thickness are shown in fig. S17. Fig. S16 indeed shows that the edge state remains present under the perpendicular magnetic field of up to 1 T.

REFERENCES

1. Zhang, J. *et al.* Raman Spectroscopy of Few-Quintuple Layer Topological Insulator Bi2Se3 Nanoplatelets. *Nano Letters* **11**, 2407-2414 (2011). DOI: 10.1021/nl200773n
2. Li, C. *et al.* A simple method to clean ligand contamination on TEM grids. *Ultramicroscopy,* **221**, 113195 (2021). DOI: 10.1016/j.ultramic.2020.113195
3. Giannozzi, P. *et al.* QUANTUM ESPRESSO: a modular and open-source software project for quantum simulations of materials. *Journal of Physics: Condensed Matter* **21**, 395502 (2009). DOI: 10.1088/0953-8984/21/39/395502
4. Van Setten, M.J. *et al.* The PseudoDojo: Training and grading a 85 element optimized norm-conserving pseudopotential table, *Computer Physics Communication* **226**, 39-54 (2018). DOI: 10.1016/j.cpc.2018.01.012
5. Perdew, J.P., Burke, K., Enzerhof, M. Generalized Gradient Approximation Made Simple, *Physics Review Letters* **78,** 3865-3868 (1996), DOI: 10.1103/PhysRevLett.77.3865
6. Sangalli, D. *et al.* Many-body perturbation theory calculations using the yambo code, *Journal of Physics: Condensed Matter* **31,** 325902 (2019), DOI: 10.1088/1361-648X/ab15d0
7. Pizzi, G. *et al.* Wannier90 as a community code: new features and applications, *Journal of Physics: Condensed Matter* **32**, 165902 (2020). DOI: 10.1088/1361-648X/ab51ff
8. Wu, QS. *et al.* WannierTools: An open-source software package for novel topological materials, *Computer Physics Communications* **224**, 405-416 (2018) DOI: 10.1016/j.cpc.2017.09.033
9. Zhang, H. J. *et al.* Topological insulators in Bi2Se3, Bi2Te3 and Sb2Te3 with a single Dirac cone on the surface. *Nature Physics* **5**, 438-442 (2009). DOI: 10.1038/nphys1270
10. Lu, H. Z. *et al.* Massive Dirac fermions and spin physics in an ultrathin film of topological insulator. *Physical Review B* **81**(11), 115407 (2010). DOI: 10.1103/PhysRevB.81.115407
11. Shan, W. Y., Lu, H. Z., Shen, S. Q. Effective continuous model for surface states and thin films of three-dimensional topological insulators. *New Journal of Physics* **12**(4), 043048 (2010). DOI: 10.1088/1367-2630/12/4/043048
12. Kane, C. L., Mele, E. J. Quantum spin Hall effect in graphene. *Physical Review Letters*, **95**(22), 226801 (2005). DOI: 10.1103/PhysRevLett.95.226801
13. Fu, L., Kane, C.L. Topological insulators with inversion symmetry. *Physical Review B* **76**(4), 045302, (2007). DOI: 10.1103/PhysRevB.76.045302
14. Kane, C. L., Mele, E. J. Z(2) topological order and the quantum spin Hall effect. *Physical Review Letters* **95,** 146802 (2005). DOI: 10.1103/PhysRevLett.95.146802
15. Zhang, S. B., Zhang, Y. Y., Shen, S. Q. Robustness of quantum spin Hall effect in an external magnetic field. *Physical Review B* **90,** 115305 (2014). DOI: 10.1103/PhysRevB.90.115305

16. Skolasinski, R. *et al.* Robust helical edge transport in quantum spin Hall quantum wells. *Physical Review B* **98,** 201404(R) (2018). DOI: 10.1103/PhysRevB.98.201404

17. Tkachov, G., Hankiewicz, E. M. Ballistic Quantum Spin Hall State and Enhanced Edge Backscattering in Strong Magnetic Fields. *Physical Review Letters* **104,** 166803 (2010). DOI: 10.1103/PhysRevLett.104.166803

18. Goldman, N., Beugeling, W., Smith, C. M. Topological phase transitions between chiral and helical spin textures in a lattice with spin-orbit coupling and a magnetic field. *Epl* **97,** 23003 (2012). DOI: 10.1209/0295-5075/97/23003

19. Knez, I., Du, R. R., Sullivan, G. Evidence for Helical Edge Modes in Inverted InAs/GaSb Quantum Wells. *Physical Review Letters* 107, 136603 (2011). DOI: 10.1103/PhysRevLett.107.136603

20. Knez, I. *et al.* Observation of Edge Transport in the Disordered Regime of Topologically Insulating InAs/GaSb Quantum Wells. *Physical Review Letters* 112, 026602 (2014). DOI: 10.1103/PhysRevLett.112.026602

21. Wang, Q., Sheng, L. Edge mode of InAs/GaSb quantum spin hall insulator in magnetic field. *Acta Phys. Sin.* **64,** 097302 (2015). DOI: 10.7498/aps.64.097302

22. Du, L. J. *et al.* Robust Helical Edge Transport in Gated InAs/GaSb Bilayers. *Physical Review Letters* **114,** 096802 (2015). DOI: 10.1103/PhysRevLett.114.096802
