# Supplementary material for: Characterization of the Edge States in Colloidal Bi2Se3 Platelets
Source: Nano Lett. 2024 Apr 16;24(17):5110–6. doi: 10.1021/acs.nanolett.3c04460 (PMC11066965; doi:10.1021/acs.nanolett.3c04460)
Supplement: Supplementary file 2 — nl3c04460_si_002.pdf [file nl3c04460_si_002.pdf]

Characterization of the edge states in colloidal Bi_2Se_3 platelets

*Jesper R. Moes[1]†, Jara F. Vliem[1]†, Pedro M. M. C. de Melo[1], Thomas C. Wigmans[1], Andrés R. Botello-Méndez[1], Rafael G. Mendes[1], Ella F. van Brenk[1], Ingmar Swart[1], Lucas Maisel Licerán[2], Henk T. C. Stoof[2], Christophe Delerue[3], Zeila Zanolli[1], *Daniel Vanmaekelbergh[1]*

Supporting figures

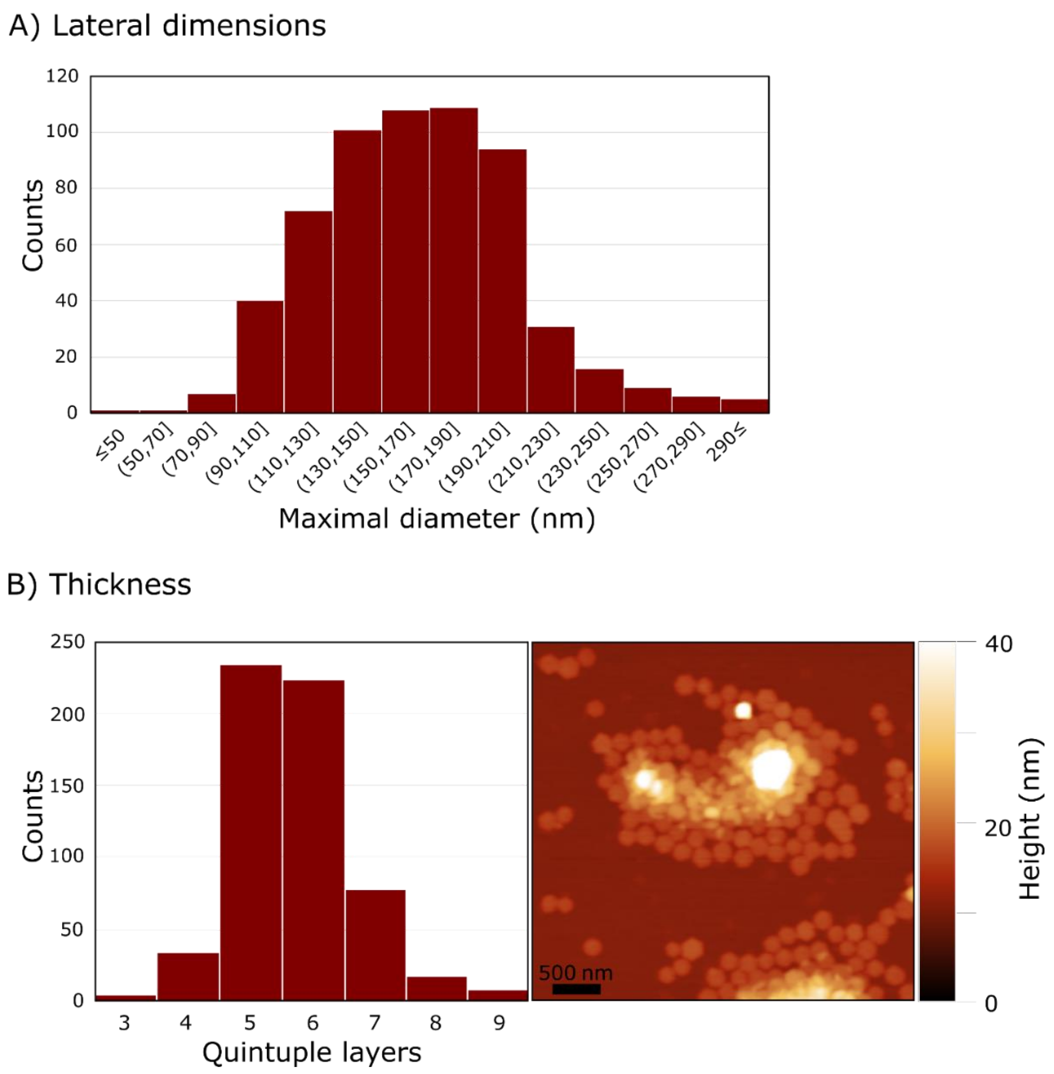

Figure S1. Lateral size and thickness of Bi_2Se_3 NPLs.

(**A**) Lateral size distribution of synthesized Bi_2Se_3 NPLs. The results were obtained from TEM images by measuring the largest corner to corner distance (i.e. maximal diameter) of 600 hexagonal NPLs of 9 different samples. An average lateral size of 166 ± 41 nm was found.

(**B**) Thickness distribution in quintuple layers, obtained by measuring 600 Bi_2Se_3 NPLs of 4 different samples with AFM. A representative AFM image is shown on the right. Because the

surface coverage of the NPLs is unknown, we did not include the effect of ligands on the measured thickness. The reported thickness may therefore be overestimated slightly.

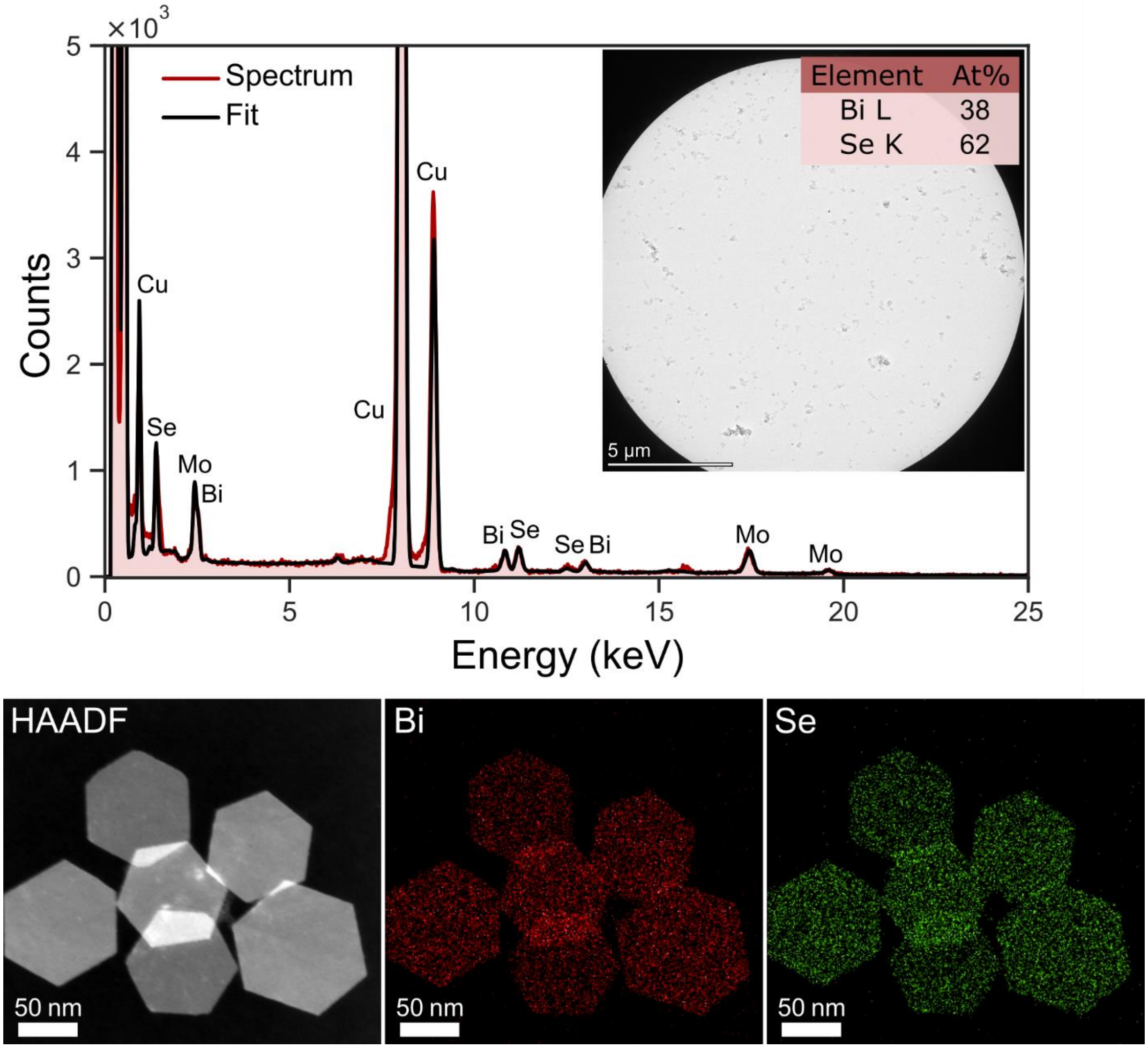

Figure S2. EDX analysis of Bi_2Se_3 NPLs.

Representative EDX spectrum, taken of the Bi_2Se_3 NPLs shown in the inset. The calculated atomic percentages (At%) of Bi and Se, averaged over 18 measurements for 6 different samples, are given in the table. Below the spectrum, a HAADF-STEM image and STEM-EDX element maps of a few Bi_2Se_3 NPLs are shown, which further confirms the formation of Bi_2Se_3 NPLs.

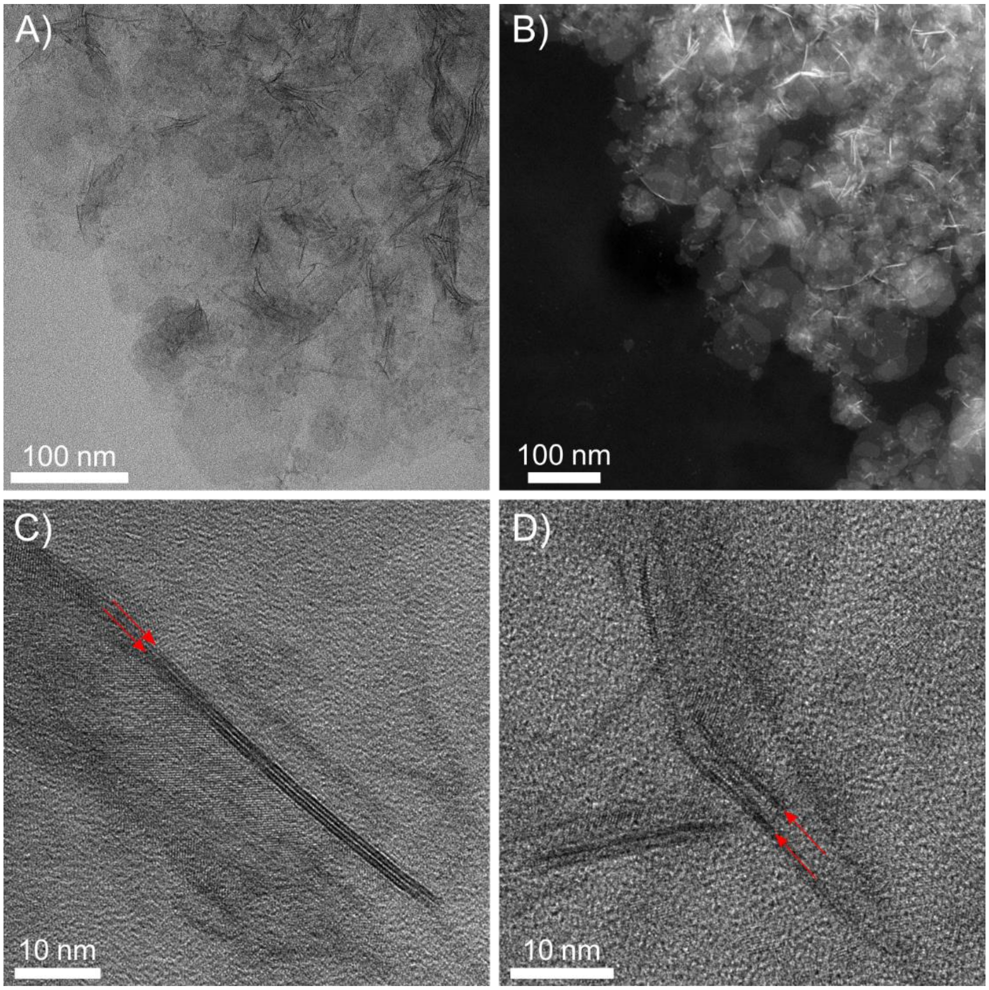

Figure S3. (HAADF-S)TEM images of ultrathin Bi_2Se_3 NPLs.

(HAADF-S)TEM images of Bi_2Se_3 NPLs (**A,B**) and the side-view of several NPLs, showing that the sample contains platelets of 2QLs (**C)** and 1QL (**D**).

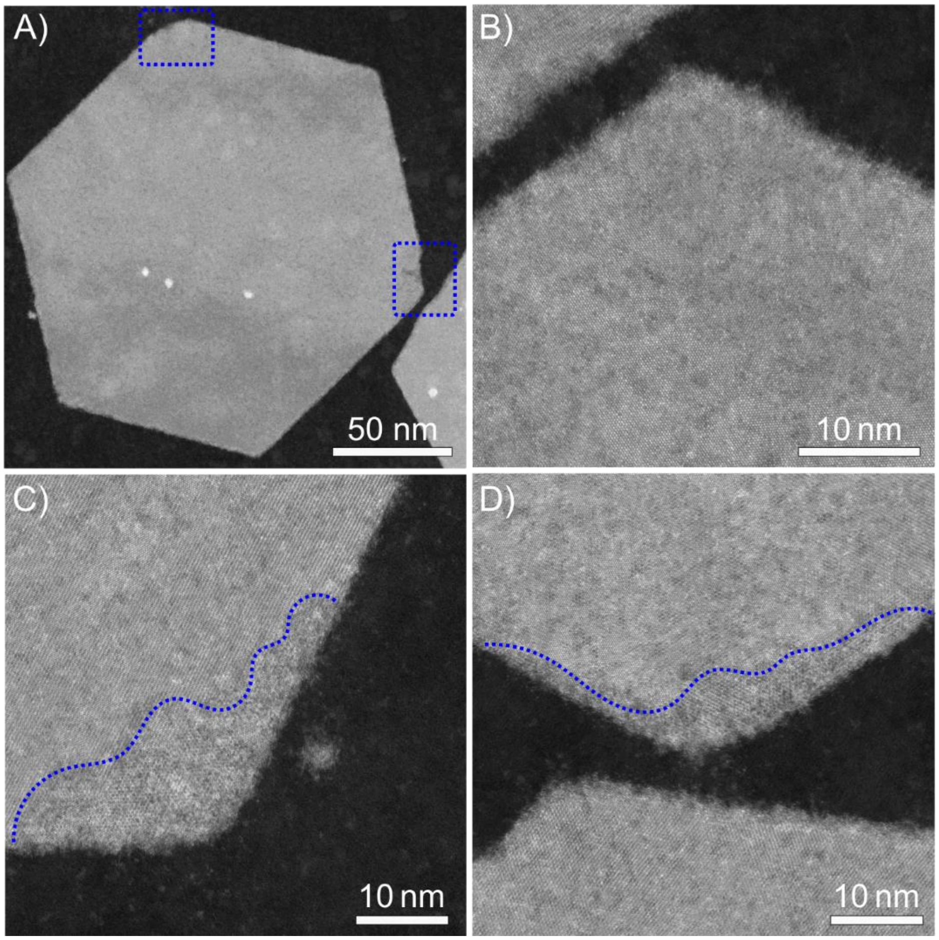

Figure S4. Impact of annealing on Bi_2Se_3 NPLs.

High resolution HAADF-STEM images of a Bi_2Se_3 NPL (**A**) and the edges/corners of several NPLs (**B-D**) after in-situ annealing for 2 hours at 393 K. The areas in (**A**), outlined in blue, show where the corners of the NPL have been affected. The blue lines in (**C**) and (**D**) are a visual indication to show the separation between areas of lower contrast (i.e. thinner regions) at the edges/corners and those with higher contrast in the center of the NPLs.

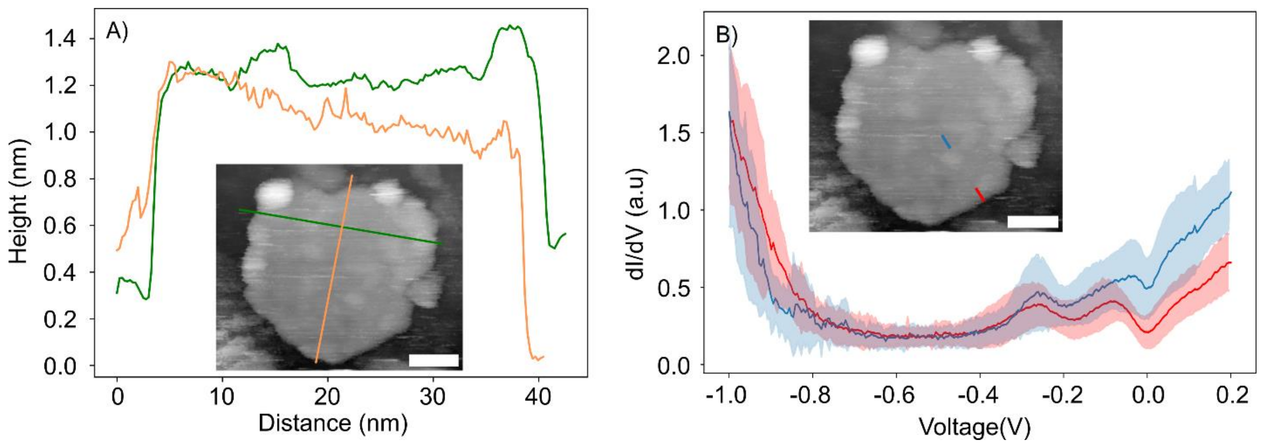

Figure S5. Characterization of the electronic states of a 1 QL thick Bi_2Se_3 platelet, in the interior and at the edge, with cryogenic scanning tunneling microscopy and spectroscopy.

(**A**) Height profile of a single platelet on a flat Au substrate along the orange and green lines shown in the inset. The diameter of the 2D sheet is about ~35 nm. The height profile shows this Bi_2Se_3 platelet consists of 1 QL.

(**B**) Scanning tunneling spectrum of the local DOS(x,y,E) taken along the blue points (see inset, region of the interior) and the red points (see inset, region of the edge). The set point in the spectroscopy is 100 pA. The interior spectrum has been averaged over 10 blue positions; the standard deviation in the DOS is indicated by a blue gloom. The red edge spectrum is averaged over the spectra taken at 10 red positions, the standard deviation is indicated by a red gloom. Scale bars are 10 nm.

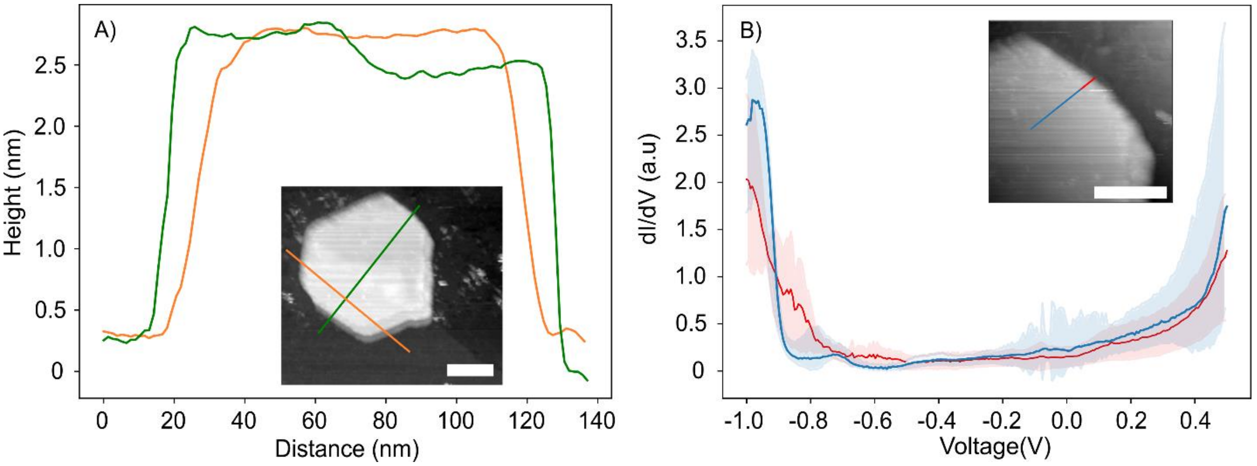

Figure S6. Characterization of the electronic states of a 3 QL thick Bi_2Se_3 platelet, in the interior and at the edge, with cryogenic scanning tunneling microscopy and spectroscopy.

(**A**) Height profile of a single platelet on a flat Au substrate along the orange and green lines shown in the inset. The diameter of the 2D sheet is about ~100 nm. The height profile shows this Bi_2Se_3 platelet consists of 3 QLs. Scale bar is 50 nm.

(**B**) Scanning tunneling spectrum of the local DOS(x,y,E) taken along the blue points (see inset, region of the interior) and the red points (see inset, region of the edge). The set point in the spectroscopy is 1 nA. The interior spectrum has been averaged over 15 blue positions; the standard deviation in the DOS is indicated by a blue gloom. The red edge spectrum is averaged over the spectra taken at 6 red positions, the standard deviation is indicated by a red gloom. Scale bar is 25 nm.

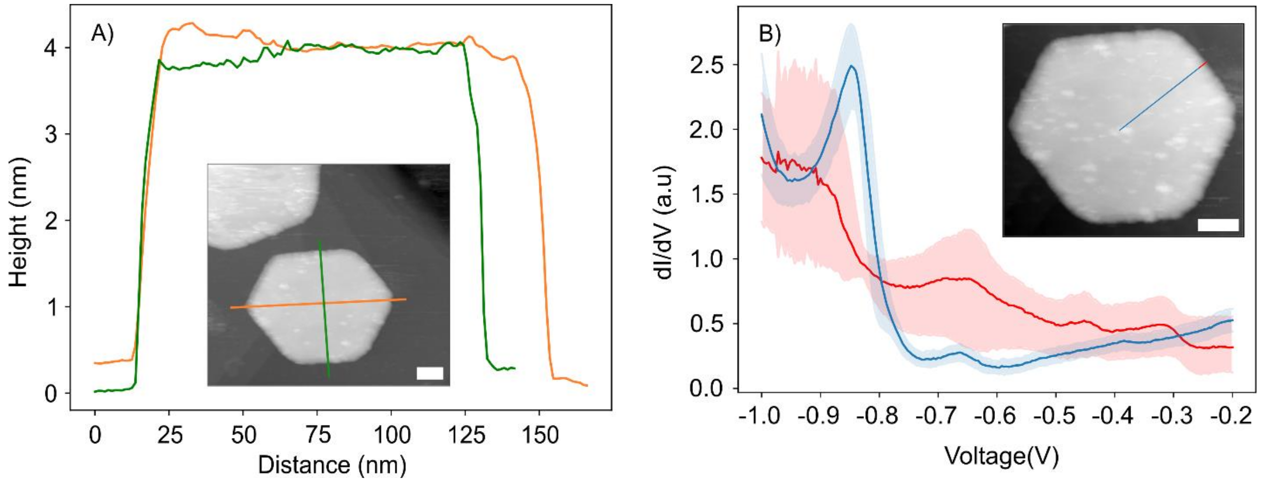

Figure S7. Characterization of the electronic states of a 4 QL thick Bi_2Se_3 platelet, in the interior and at the edge, with cryogenic scanning tunneling microscopy and spectroscopy.

(**A**) Height profile of a single platelet on a flat Au substrate along the orange and green lines shown in the inset. The diameter of the 2D sheet is about 125 nm. The height profile shows this Bi_2Se_3 platelet consists of 4 QLs.

(**B**) Scanning tunneling spectrum of the local DOS(x,y,E) taken along the blue points (see inset, region of the interior) and the red points (see inset, region of the edge). The set point in the spectroscopy is 1nA. The interior spectrum has been averaged over 25 blue positions; the standard deviation in the DOS is indicated by a blue gloom. The red edge spectrum is averaged over the spectra taken at 5 red positions, the standard deviation is indicated by a red gloom. Scale bars are 25nm.

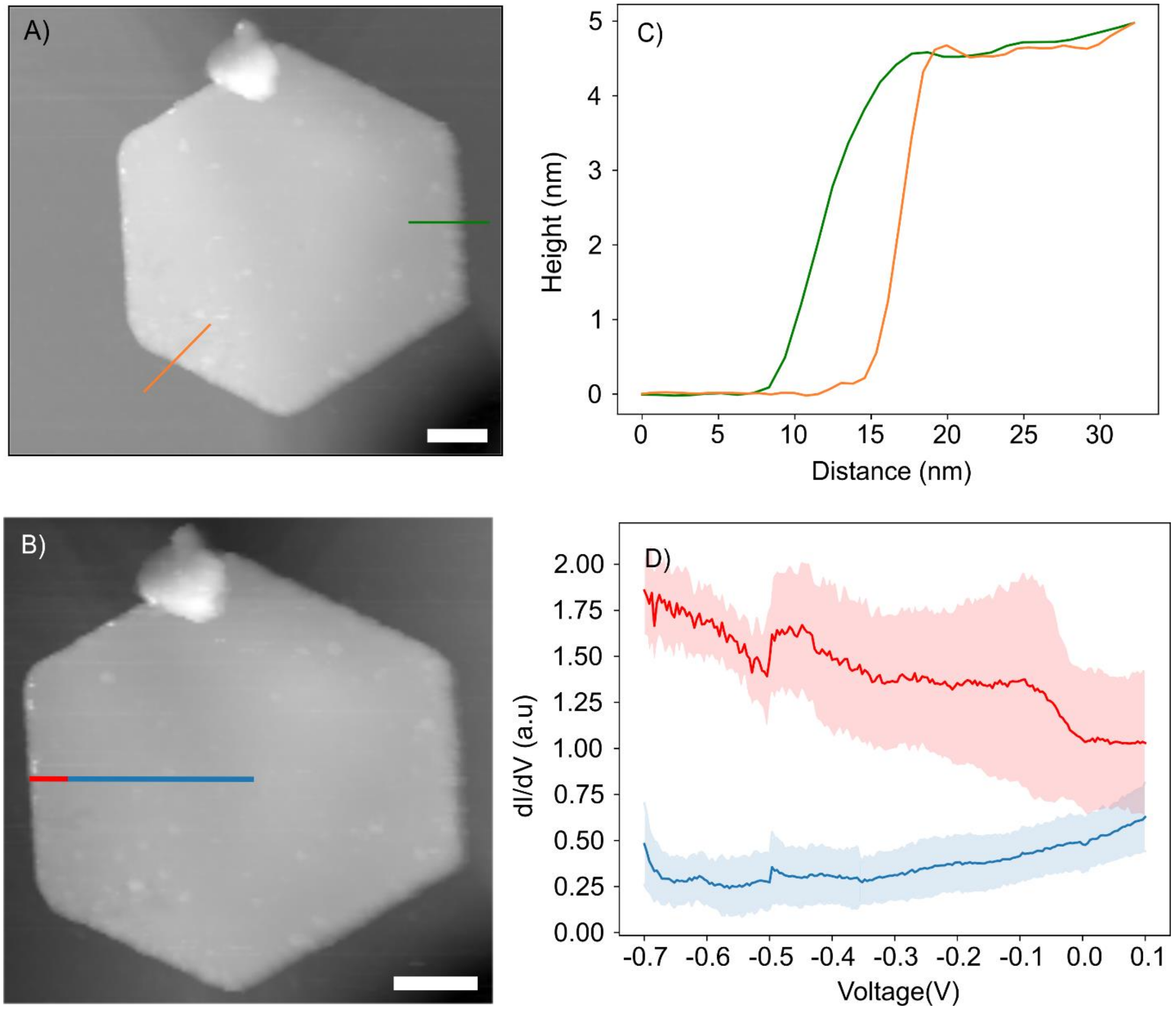

Figure S8. Characterization of the electronic states of a 5 QL thick Bi_2Se_3 platelet, in the interior and at the edge, with cryogenic scanning tunneling microscopy and spectroscopy.

(**A**) Scanning tunneling constant current image showing a Bi_2Se_3 platelet on a flat Au substrate along the orange and green lines, height profiles are taken which are shown in fig S.3c.

(**B**) Scanning tunneling constant current image showing the same Bi_2Se_3 platelet; the blue and red lines indicate the positions of tunneling spectroscopy (fig S.8D).

(**C**) Height profile of the same single platelet shown in fig S3a. The diameter of the 2D sheet is about 125nm. The height profile shows this Bi_2Se_3 platelet consists of 5 quintuple layers (QLs).

(**D**) Scanning tunneling spectrum of the local DOS(x,y,E) taken along the blue points (see fig S.3b, region of the interior) and the red points (see fig S3b, region of the edge). The set point in the spectroscopy is 1nA. Please, notice the smaller bias range to focus on the bias region with increased intensity of the edge. The interior spectrum has been averaged over 20 blue positions; the standard deviation in the DOS is indicated by a blue gloom. The red edge spectrum is averaged over the spectra taken at 6 red positions, the standard deviation is indicated by a red gloom. Scale bars are 25 nm.

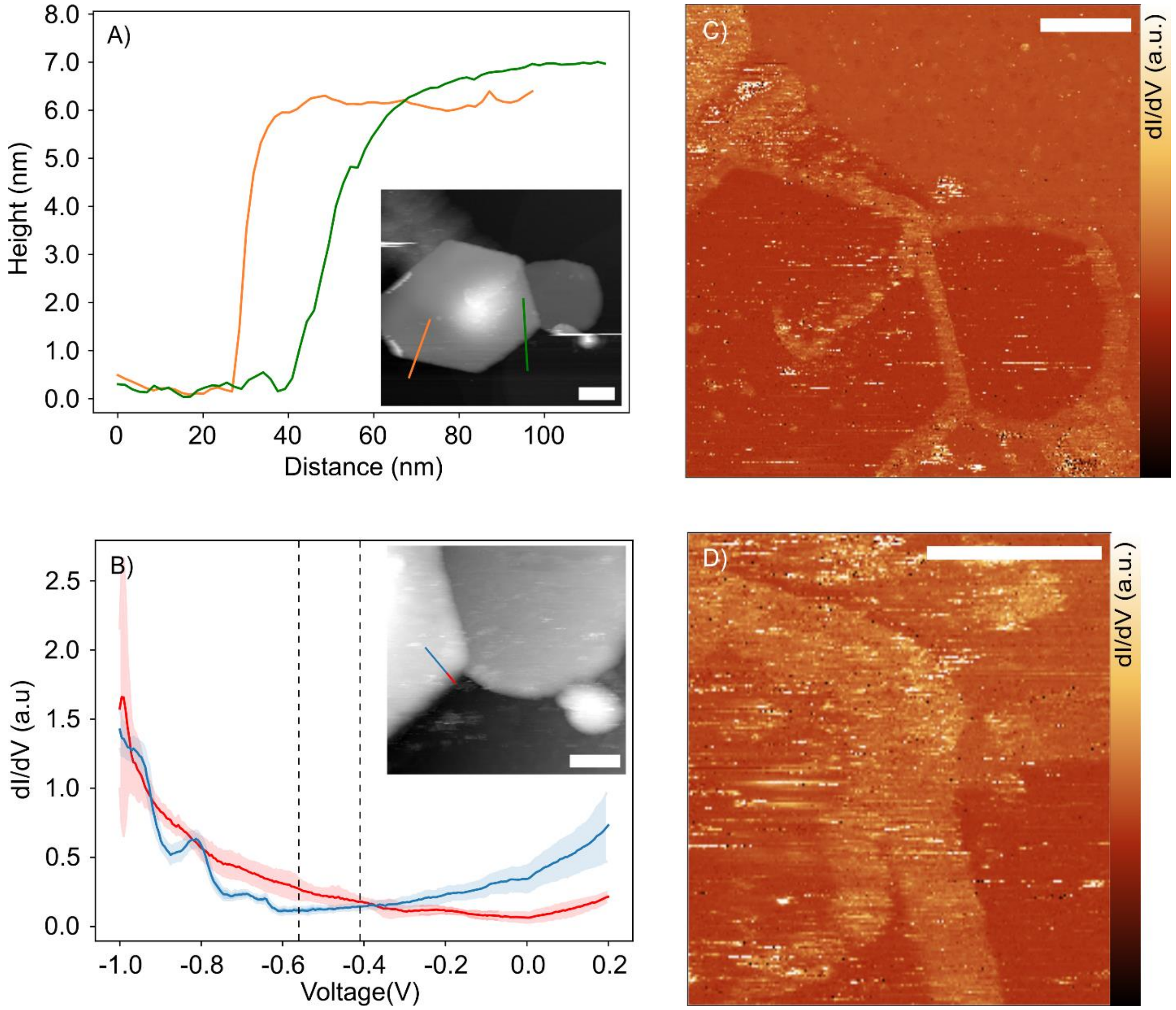

Figure S9. Characterization of the electronic states of a 6 QL thick Bi_2Se_3 platelet, in the interior and at the edge, with cryogenic scanning tunneling microscopy and spectroscopy.

(**A**) Height profile of a single platelet on a flat Au substrate along the orange and green lines shown in the inset. Also shown in the inset is the second smaller platelet attached to the bigger platelet. The diameter of the larger 2D sheet is about 200 nm. The height profile shows this Bi_2Se_3 platelet consists of 6 quintuple layers (QLs). Scalebar is 50 nm.

(**B**) Scanning tunneling spectrum of the local DOS(x,y,E) taken along the blue points (see inset, region of the interior) and the red points (see inset, region of the edge). The set point in the

spectroscopy is 1nA. The interior spectrum has been averaged over 5 blue positions; the standard deviation in the DOS is indicated by a blue gloom. The red edge spectrum is averaged over the spectra taken at 6 red positions, the standard deviation is indicated by a red gloom. Scalebar is 25nm.

(**C**) LDOS(x,y) map of the Bi_2Se_3 platelet acquired at a bias V of -0.41 V at which the DOS(x,y,E) has a higher intensity than the interior. From the top right to the center of the larger platelet an increase intensity can be seen coming from a screw dislocation in the platelet. On the right there is the increased intensity seen on the smaller 4 QL platelet discussed in figs. 3 and S2. Scale bar is 50 nm.

(**D**) LDOS(x,y) map of the Bi_2Se_3 platelet acquired at a bias V of -0.56 V at which the DOS(x,y,E) at the edge is higher than in the interior; a 8 nm wide band of high density of states follows the edge of the 6 QL crystal on the left, the 4 QL crystal on the right and the additional edge in the bottom left arising from a screw dislocation. Scalebar is 25 nm.

We observe here that the edge state binds to the screw dislocation. A similar observation has been made in the case of a three-dimensional Bi-crystal, which brought the authors to conclude that Bi belongs to the class of strong topological insulators, not to the class of crystalline higher-order topological insulators (*1*).

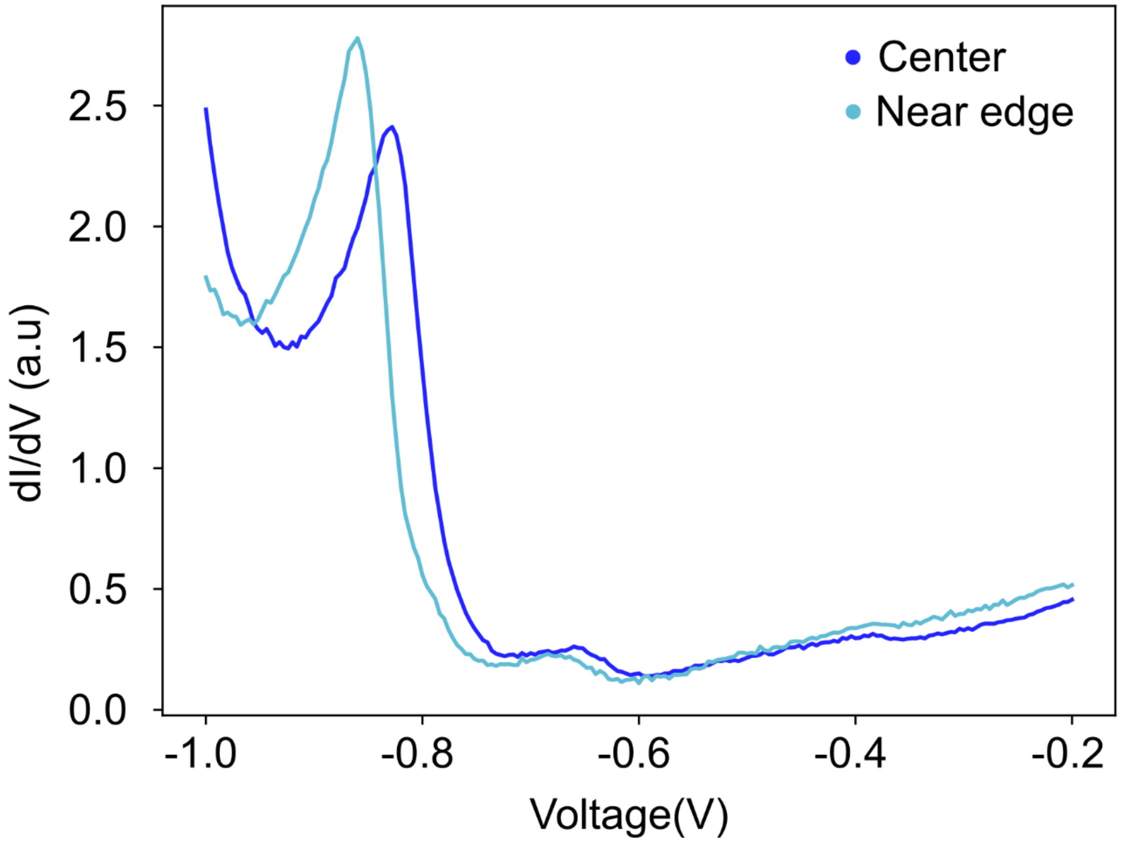

Figure S10. Scanning tunneling spectrum of the local DOS(x,y,E), taken on a 5 QL thick NPL.

The dark blue spectrum is an average of 10 positions in the center of the interior of the platelet. The light blue spectrum is an average of 3 positions less than 1nm away from the confirmed edge state. The setpoint in the spectroscopy is 1 nA.

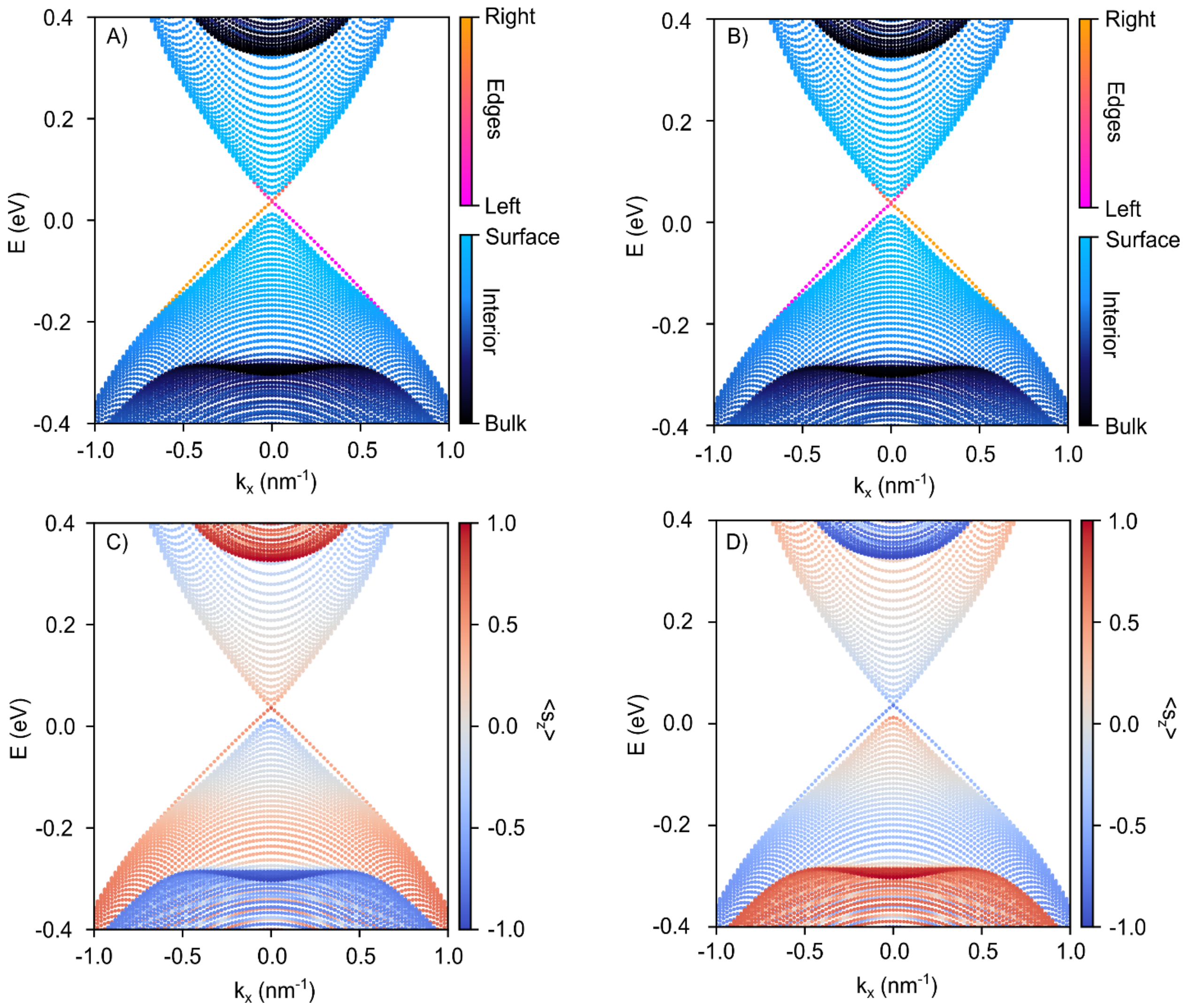

Figure S11. Energy spectrum of a 100-nm-wide ribbon of 4 QL thickness as calculated from the 8-band model.

(**A,B)** The surface-bulk character of the interior bands and the localization of the edge states for the two uncoupled 4×4 subspaces. **(C,D)** The projection on the z-axis of the spin polarization averaged over the z-direction (perpendicular to the platelet) for the two uncoupled subspaces. For each spin direction in the first subspace with a certain velocity and localized at one edge, there is an opposite spin state in the second subspace, whose velocity is opposite and which is localized at the other edge. Hence, putting together both subspaces gives the usual QSH picture.

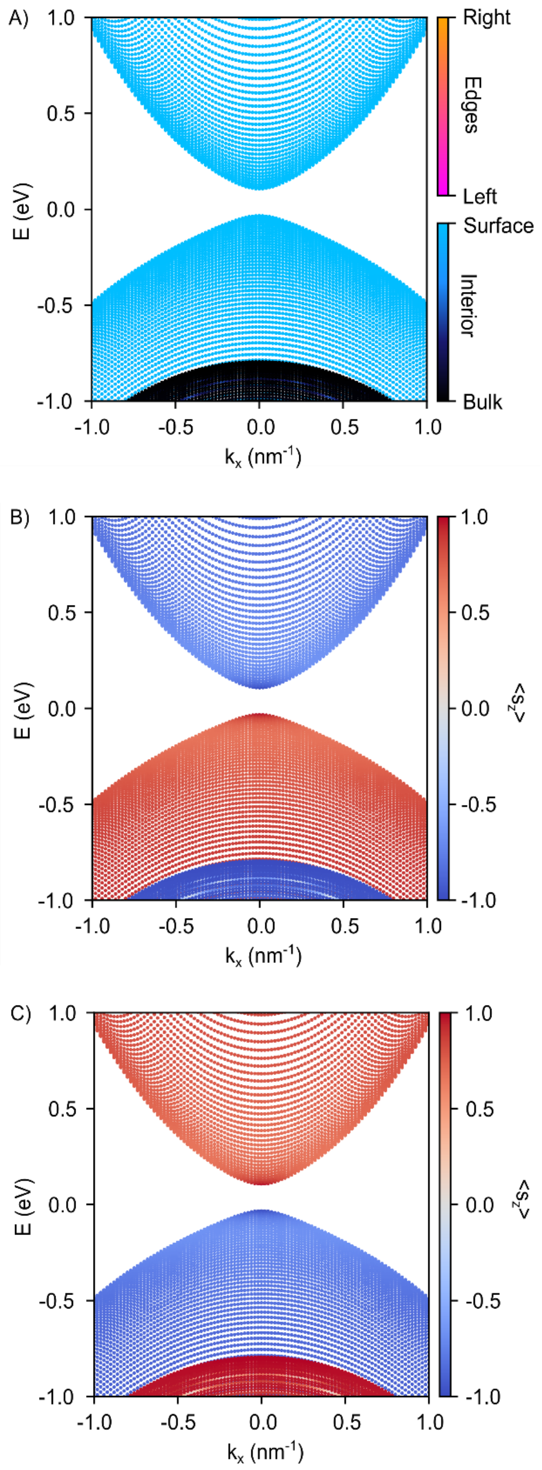

Figure S12. Energy spectrum of a 100 nm wide ribbon of 2 QL thickness as calculated from the 8-band model.

In this case there are no edge states as the Z_2 invariant is trivial. **(A)** The surface-bulk character of the states is identical in the two subspaces. (**B,C)** The spin of both subspaces is reversed as a consequence of TRS.

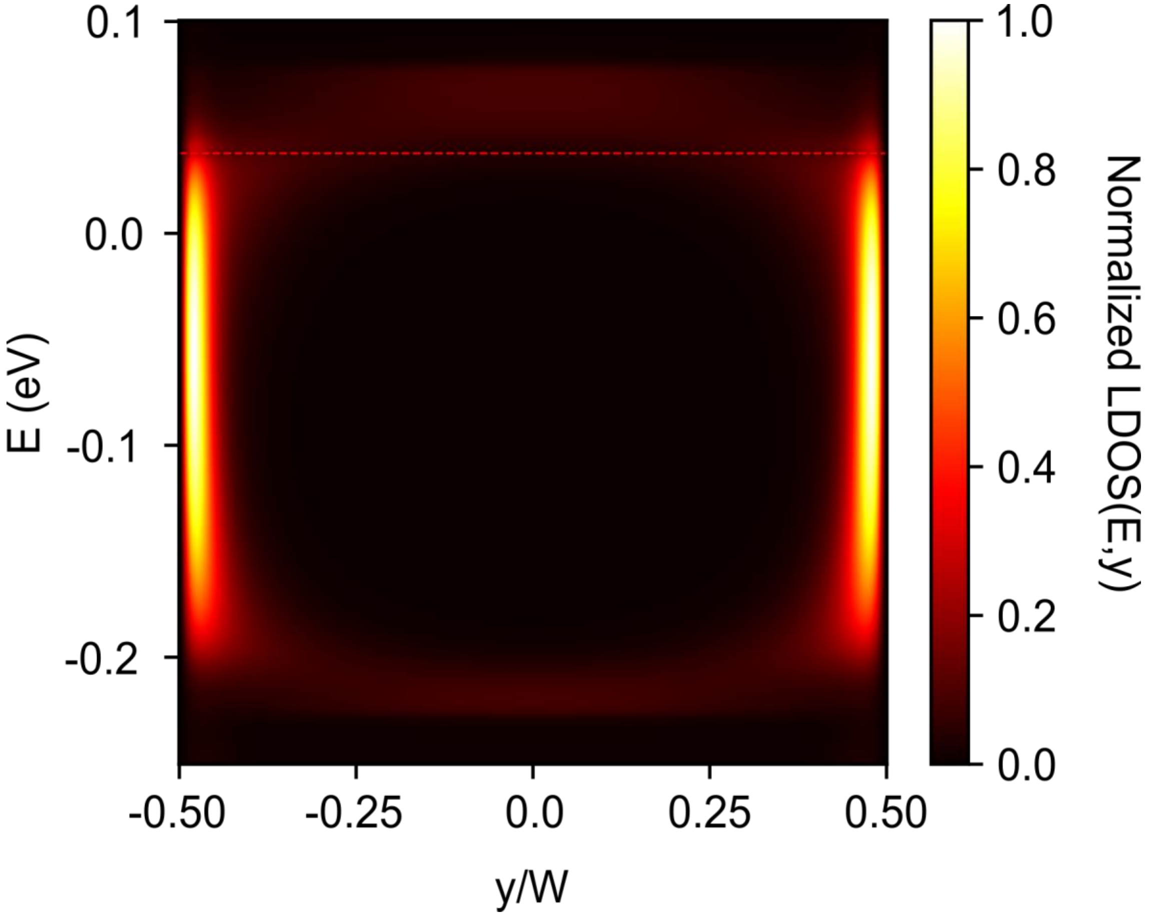

Figure S13. LDOS of the topological edge states on a ribbon of width W = 100 nm and 4 QLs in thickness.

The states span an energy range of around 200 meV and penetrate about 9 nm into the sample at each edge. The red dashed line signals the position of the Dirac point.

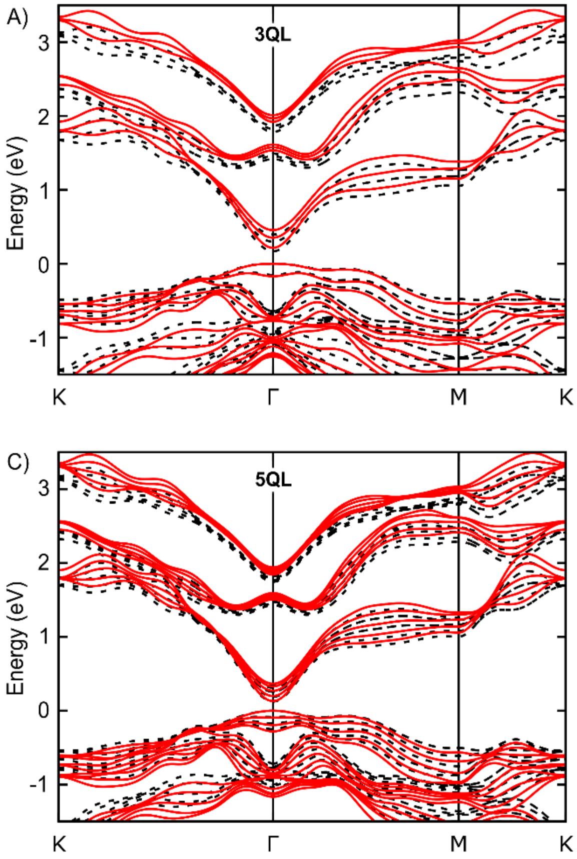

Figure S14. Electronic band structures computed with DFT (black dashed lines) and GW (red full lines) for the 3−6 QL slabs.

The band structures for 3, 4, 5, 6 QL slabs of infinite lateral size are shown in figures **A, B, C, D**, respectively. The small band-gap opening at Γ in the 3QL slab reduces with increasing number of QL. We notice that the effective mass of the electrons is renormalized by the self-energy in the GW bands.

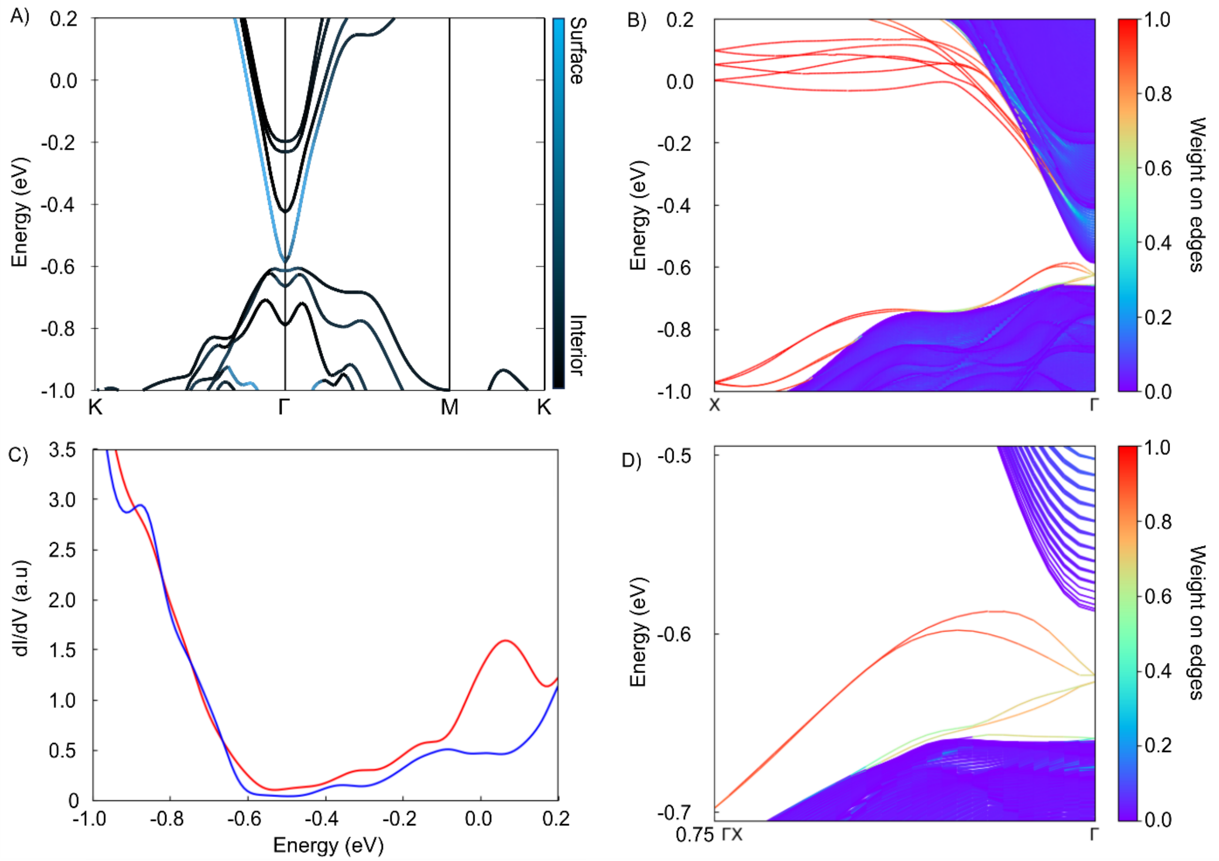

Figure S15. Theoretical analysis of the interior and surface for a 2D infinite crystal, and edge states for 2D Bi_2Se_3 ribbons, 4 QLs in thickness and 100 nm in width, based on GW-TB calculations.

(**A**) Electronic band structure of a two-dimensional Bi_2Se_3 crystal, 4 QLs in thickness, computed within the GW approximation. The atomic structure is partly relaxed using first-principle DFT simulations, but some strain remains. Interior states are indicated in black, surface states in light blue. (**B**) Band structure along the **Γ**-X line for a 4QL Bi_2Se_3 ribbon of 100 nm in width. The color indicates the weight of the wavefunction on the regions of 2 nm from the edges. The bands of the interior region are in blue, the states situated at and close to the edges are in yellow-red. (**C**) The density of states for a ribbon of 4 QLs in thickness and 100 nm in width. The states of the interior region (blue) include the top of the valence band, the hybridized surface states and

the conduction states. The LDOS in the edge region is in red. (**D**) Detail of the band structure shown in (B) near the Fermi energy around the **Γ point.**

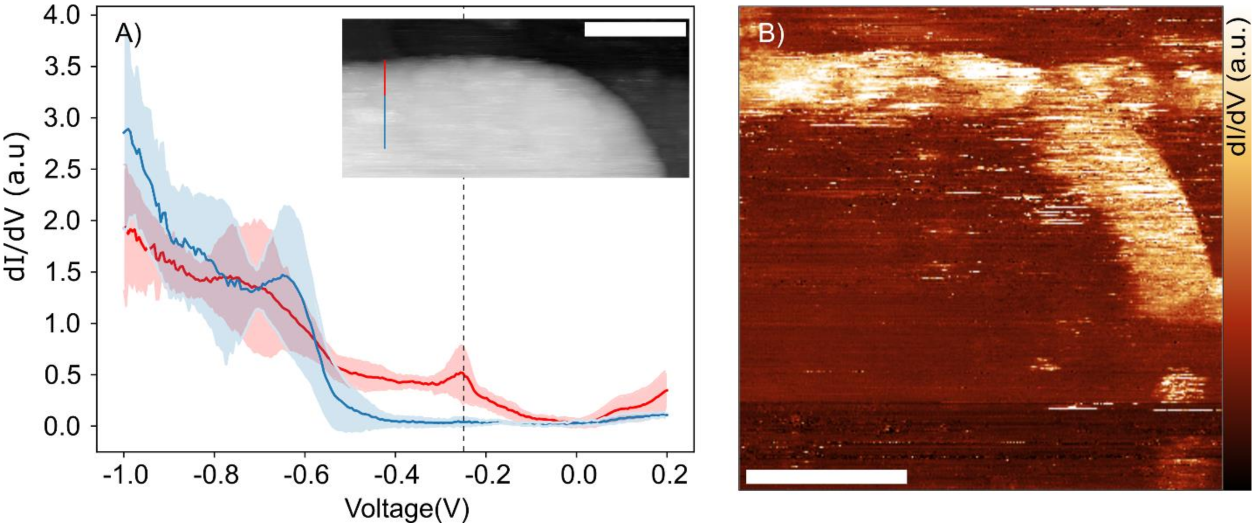

Figure S16. Characterization of the electronic states of a 4 QL Bi_2Se_3 platelet under a perpendicular magnetic field of 1 T.

The platelet is the same as that investigated in the absence of a magnetic field, see Fig. 2. (**A**) Scanning tunneling spectrum of the LDOS(E,x,y) averaged 6 positions on the blue line, representing the interior area of the platelet. The red spectrum is taken over 8 positions over the red line and represents the edge state. The set point in the spectroscopy is 1 nA. (**B**) Map of the LDOS(x,y,E) acquired for the upper-right corner of the platelet, at a voltage of -0.25 V to highlight the edge state. The width of the edge state is about 10 nm. The scale bar is 20 nm.

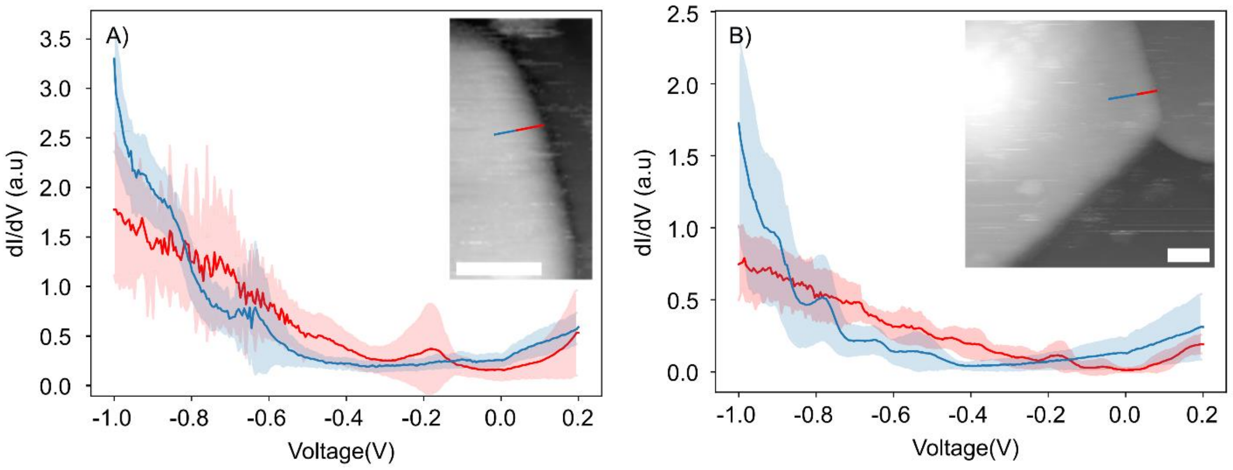

Figure S17. Characterization of the electronic states of Bi_2Se_3 platelets, in the interior and at the edge, with cryogenic scanning tunneling microscopy and spectroscopy at 0.5 T.

The red data is acquired on the interior of the NPL (shown in the inset) with the standard deviation presented as a red gloom. The blue data on the edge of the NPL (shown in the inset) with the standard deviation presented as a blue gloom.

(**A**) Scanning tunneling spectrum of the local DOS(x,y,E) taken on a 4 QL thick NPL, along the blue points (see inset, region of the interior) and the red points (see inset, region of the edge). The set point in the spectroscopy is 1 nA. The interior spectrum has been averaged over 6 blue positions; the standard deviation in the DOS is indicated by a blue gloom. The red edge spectrum is averaged over the spectra taken at 13 red positions, the standard deviation is indicated by a red gloom.

(**B**) Scanning tunneling spectrum of the local DOS(x,y,E) taken, on a 6 QL thick NPL, along the blue points (see inset, region of the interior) and the red points (see inset, region of the edge). The set point in the spectroscopy is 1nA. The interior spectrum has been averaged over 8 blue positions; the standard deviation in the DOS is indicated by a blue gloom. The red edge

spectrum is averaged over the spectra taken at 6 red positions, the standard deviation is indicated by a red gloom. Scale bars are 20 nm.

REFERENCES

1. Nayak, A. K. *et al*. Resolving the topological classification of bismuth with topological defects. *Science advances* **5**(11), eaax6996 (2019). DOI: 10.1126/sciadv.aax6996
