# Supplementary material for: Characterization of the Edge States in Colloidal Bi2Se3 Platelets
Source: Nano Lett. 2024 Apr 16;24(17):5110–6. doi: 10.1021/acs.nanolett.3c04460 (PMC11066965; doi:10.1021/acs.nanolett.3c04460)
Supplement: Supplementary file 3 — nl3c04460_si_003.pdf [file nl3c04460_si_003.pdf]

Characterization of the edge states in colloidal Bi_2Se_3 platelets

*Jesper R. Moes[1]†, Jara F. Vliem[1]†, Pedro M. M. C. de Melo[1], Thomas C. Wigmans[1], Andrés R. Botello-Méndez[1], Rafael G. Mendes[1], Ella F. van Brenk[1], Ingmar Swart[1], Lucas Maisel Licerán[2], Henk T. C. Stoof[2], Christophe Delerue[3], Zeila Zanolli[1], *Daniel Vanmaekelbergh[1]*

Supporting tables

Table S1: Cell lattice parameters for the several Bi_2Se_3 systems, obtained after performing optimization using spin-orbit coupling. The selenium-selenium distance in the internal ('inner') QL and surface ('outer') QL is reported as well.

System	a [Å]	d_{Se-Se} [Å] (inner-outer)
1QL	4.171	7.058
2QL	4.185	7.033
3QL	4.191	7.022-7.024
4QL	4.1932	7.0179-7.021
5QL	4.1946	7.0156-7.0199
6QL	4.211	6.9895-7.003
Bulk	4.192	7.0202

Table S2: Computed $\mathbb{Z}_2$ invariants using Wannier90 and $\mathbb{Z}_2$pack for 1 to 6 QL thick Bi_2Se_3 slabs and bulk Bi_2Se_3 displaying the topological nature of the system with thickness ≥ 4 QL.

System	DFT $\mathbb{Z}_2$	G_0W_0 $\mathbb{Z}_2$
1QL	0	0
2QL	0	0
3QL	0	0
4QL	1	1
5QL	1	1
6QL	1	1
Bulk	1	1

Table S3: Calculated bandgaps using DFT, including G_0W_0 correction, for Bi_2Se_3 slabs of 1 to 6 QLs as well as bulk Bi_2Se_3.

System	DFT gap [meV]	G_0W_0 correction [meV]
1QL	755	234
2QL	65	105
3QL	8	44
4QL	4(inverted)	54 (inverted)
5QL	2(inverted)	35(inverted)
6QL	3(inverted)	24(inverted)
Bulk	406 (inverted)	43 (inverted)
